# Inducible Nitric Oxide Synthase Is a Key Host Factor for *Toxoplasma* GRA15-Dependent Disruption of the Gamma Interferon-Induced Antiparasitic Human Response

**DOI:** 10.1128/mBio.01738-18

**Published:** 2018-10-09

**Authors:** Hironori Bando, Youngae Lee, Naoya Sakaguchi, Ariel Pradipta, Ji Su Ma, Shun Tanaka, Yihong Cai, Jianfa Liu, Jilong Shen, Yoshifumi Nishikawa, Miwa Sasai, Masahiro Yamamoto

**Affiliations:** a Department of Immunoparasitology, Research Institute for Microbial Diseases, Osaka, Japan; b Laboratory of Immunoparasitology, WPI Immunology Frontier Research Center, Osaka University, Osaka, Japan; c Institute of Zoonoses, The Key Laboratory of Pathogen Biology, The Key Laboratory of Zoonoses, Anhui Medical University, Hefei, Anhui, China; d Department of Pathology and Pathogenic Biology, Medical College of Ningbo University, Ningbo City, China; e National Research Center for Protozoan Diseases, Obihiro University of Agriculture and Veterinary Medicine, Obihiro, Hokkaido, Japan; University of New Mexico; Stanford University

**Keywords:** *Toxoplasma gondii*, cell-autonomous immunity, host-parasite interaction, human immunology, immune suppression, interferon

## Abstract

Although *Toxoplasma* virulence mechanisms targeting gamma interferon (IFN-γ)-induced cell-autonomous antiparasitic immunity have been extensively characterized in mice, the virulence mechanisms in humans remain uncertain, partly because cell-autonomous immune responses against *Toxoplasma* differ markedly between mice and humans. Despite the identification of inducible nitric oxide synthase (iNOS) as an anti-*Toxoplasma* host factor in mice, here we show that iNOS in humans is a pro-*Toxoplasma* host factor that promotes the growth of the parasite. The GRA15 *Toxoplasma* effector-dependent disarmament of IFN-γ-induced parasite growth inhibition was evident when parasite-infected monocytes were cocultured with hepatocytes. Interleukin-1β (IL-1β), produced from monocytes in a manner dependent on GRA15 and the host’s NLRP3 inflammasome, combined with IFN-γ to strongly stimulate iNOS expression in hepatocytes; this dramatically reduced the levels of indole 2,3-dioxygenase 1 (IDO1), a critically important IFN-γ-inducible anti-*Toxoplasma* protein in humans, thus allowing parasite growth. Taking the data together, *Toxoplasma* utilizes human iNOS to antagonize IFN-γ-induced IDO1-mediated cell-autonomous immunity via its GRA15 virulence factor.

## Abstract (precis)

*Toxoplasma,* an important intracellular parasite of humans and animals, causes life-threatening toxoplasmosis in immunocompromised individuals. Gamma interferon (IFN-γ) is produced in the host to inhibit the proliferation of this parasite and eventually cause its death. Unlike mouse disease models, which involve well-characterized virulence strategies that are used by *Toxoplasma* to suppress IFN-γ-dependent immunity, the strategies used by *Toxoplasma* in humans remain unclear. Here, we show that GRA15, a *Toxoplasma* effector protein, suppresses the IFN-γ-induced indole-2,3-dioxygenase 1-dependent antiparasite immune response in human cells. Because NLRP3-dependent production of IL-1β and nitric oxide (NO) in *Toxoplasma*-infected human cells is involved in the GRA15-dependent virulence mechanism, blocking NO or IL-1β production in the host could represent a novel therapeutic approach for treating human toxoplasmosis.

## INTRODUCTION

Toxoplasma gondii is an obligatory protozoan parasite that can infect nearly all warm-blooded animals, including humans (1, 2). It is estimated that one-third of the world’s human population is infected with T. gondii; notably, most infections are asymptomatic. T. gondii, however, also causes toxoplasmosis in immunocompromised individuals; the clinical signs of toxoplasmosis comprise encephalitis, hepatitis, and myocarditis. Individuals with increased susceptibility to toxoplasmosis include those with AIDS, those undergoing chemotherapy, fetuses with congenital diseases, and newborn babies of women who initially contracted the infection during pregnancy (3–5). T. gondii is ranked among the top five human pathogens that cause economic loss and life impairment via food-borne illness in the United States (6). Thus, T. gondii is an important pathogen of both humans and animals.

T. gondii secretes various effector molecules into host cells upon infection to promote efficient parasite growth and dissemination *in vivo* (7, 8). The effector mechanisms used by the parasite to subvert host immune responses have been extensively analyzed in mouse models. The proteins ROP5, ROP16, ROP17, ROP18, GRA7, and TgIST are secreted from rhoptries or dense granules to suppress anti-T. gondii cell-autonomous immune responses; this results in increased parasite virulence in mice (9–19). GRA6, a dense granule protein, activates the host transcription factor NFAT4 to induce chemokines and recruit neutrophils to sites of infection, thereby promoting parasite dissemination and maximizing parasite virulence (20). GRA15, another dense granule protein, is secreted into host cells to activate another host transcription factor, NF-κB, in both mouse and human cells (21–23). Similarly to GRA6, GRA15 activates host immune responses and mediates interleukin-1 (IL-1) production via activation of the NLRP3 inflammasome (23, 24). Lack of GRA15 in T. gondii parasites promotes *in vivo* parasite proliferation in mice (22). Given that GRA15-deficient T. gondii is more virulent than wild-type (WT) T. gondii in mice, GRA15 might assist host survival by limiting parasite replication; hence, it may play an antiparasitic role in mice (19, 22). However, the significance of GRA15 as a virulence factor in humans is not well understood.

The mechanisms underlying host resistance to T. gondii rely on innate and adaptive immunity and involve various immune/nonimmune cells and cytokines. Among these contributing factors, interferon-γ (IFN-γ), which is the most important host cytokine that targets T. gondii, is largely produced by _CD4_^+^ T cells and natural killer cells; it stimulates cell-autonomous responses in immune cells, including macrophages and dendritic cells, or nonimmune cells (e.g., fibroblasts) (25). IFN-γ activates the STAT1 transcription factor and induces the expression of hundreds of genes (26). IFN-γ-inducible GTPases and nitric oxide (NO) mediate parasite clearance and growth inhibition in mice, respectively (27); however, they may not play major roles in these processes in humans (28–30). IFN-γ-dependent nutrient deprivation and cell death have been established as anti-T. gondii responses in human cells (31, 32). IFN-γ stimulates the expression of indoleamine 2,3-dioxygenase (IDO) to degrade tryptophan, an essential nutritional amino acid for the intracellular growth of T. gondii in human cells (33, 34). Thus, although IFN-γ is important for responses against T. gondii in both humans and mice, the IFN-γ-inducible effector mechanisms differ greatly between these two hosts.

The anti-T. gondii role for inducible NO synthase (iNOS), an IFN-γ-inducible protein, has been established in mice (35). Deletion or pharmacological inhibition of the iNOS gene in mouse macrophages results in profoundly reduced NO production in response to IFN-γ, along with concomitant parasite growth (28, 36). However, blocking iNOS activity does not affect the IFN-γ-induced antiparasite response in human macrophages or monocytes (28). Thus, although iNOS may play different roles in mice and humans, its precise role in the IFN-γ-mediated interplay between humans and T. gondii remains unknown.

We describe here a novel virulence strategy for T. gondii whereby the pathogen utilizes the GRA15 effector protein and the iNOS host cofactor to suppress the IFN-γ-induced IDO-dependent cell-autonomous immune response in human cells.

## RESULTS

### GRA15 promotes T. gondii growth in cocultures of THP-1 and Huh7 cells in the presence of IFN-γ.

**FIG 1:**
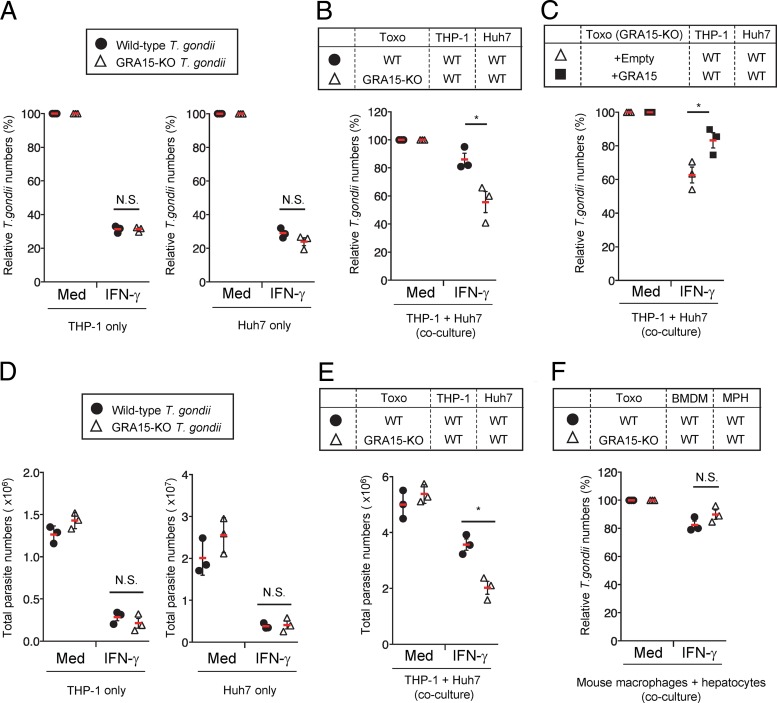
GRA15 suppresses IFN-γ-induced parasite reduction under THP-1/Huh7 coculture conditions. (A) THP-1 or Huh7 cells were left untreated (medium only [Med]) or treated with IFN-γ for 24 h and then infected with Pru T. gondii. The parasite survival rates were measured by luciferase assay. (B) THP-1 cells were infected with WT or GRA15-KO Pru T. gondii. The infected THP-1 cells were cocultured with Huh7 cells and then left untreated or treated with IFN-γ. The parasite survival rates were measured by luciferase assay. (C) THP-1 cells were infected with GRA15-KO Pru T. gondii stably expressing GRA15 or empty vectors. The infected THP-1 cells were cocultured with Huh7 cells and then left untreated or treated with IFN-γ. The parasite survival rates were measured by luciferase assay. (D) THP-1 or Huh7 cells were left untreated or treated with IFN-γ for 24 h and then infected with wild-type or GRA15-KO Pru T. gondii. The parasite numbers were calculated by quantification of the amount of genomic DNA of the *sag1* gene, which was measured by qPCR, using the standard curve shown in Fig. S1F. (E) THP-1 cells were infected with WT or GRA15-KO Pru T. gondii. The infected THP-1 cells were cocultured with Huh7 cells and then left untreated or treated with IFN-γ. The numbers were calculated by quantification of the amount of genomic DNA of the *sag1* gene, which was measured by qPCR, using the standard curve shown in Fig. S1F. (F) BMDMs were infected with WT or GRA15-KO Pru T. gondii. The infected BMDMs were cocultured with primary mouse hepatocytes and then left untreated or treated with IFN-γ. The parasite survival rates were measured by luciferase assay. Indicated values represent means ± standard deviations (SD) (three biological replicates per group from three independent experiments) (panels A to F). *, *P* < 0.05; N.S., not significant (Student's *t* test).

The significance of GRA15 as a virulence factor in humans is unclear; therefore, we explored the use of clustered regularly interspaced short palindromic repeat (CRISPR)/Cas9 genome-edited GRA15-deficient (i.e., GRA15 knockout [GRA15-KO]) parasites to clarify its role (see Fig. S1A and B in the supplemental material). There were no differences in the levels of growth of wild-type and GRA15-KO T. gondii in various IFN-γ-prestimulated or IFN-γ-poststimulated human cell lines, including the THP-1 acute monocytic leukemia cell line and Huh7 hepatoma cell line (Fig. 1A and data not shown) (see also Fig. S1D). In addition, GRA15-KO T. gondii proliferated in a manner similar to that seen with wild-type parasites in THP-1 and Huh7 cells (Fig. S1C). During parasite dissemination *in vivo*, T. gondii infects _CD11b_^+^ cells and translocates itself from infected sites to multiple organs (20, 37) where T. gondii-infected monocytes can interact with various tissue cells. To mimic this *in vivo* monocyte-hepatocyte interaction using an *in vitro* design, various coculture models have been developed with human immune cell lines and tissue cell lines; one of these models utilizes the THP-1 and Huh7 cell lines (38). Thus, we tested cocultures of THP-1 cells and Huh7 cells, in which THP-1 cells were infected with T. gondii for 24 h; subsequently, infected THP-1 cells and culture supernatants were seeded on Huh7 cells, with or without IFN-γ. These cocultures were incubated for a further 48 h; numbers of parasites were then compared (Fig. 1B). Surprisingly, the GRA15-KO parasite-infected THP-1/Huh7 coculture condition resulted in a significant reduction in the numbers of parasites compared with the wild-type parasite-infected condition (Fig. 1B). To test whether the reduction of the number of GRA15-KO parasites in the THP-1/Huh7 coculture model was due to the lack of GRA15 in the parasites, we complemented GRA15 in the GRA15-KO parasites (Fig. S1E) and then analyzed parasite growth under the coculture conditions (Fig. 1C). The numbers of GRA15-KO parasites complemented with GRA15 significantly increased in the IFN-γ-stimulated THP-1/Huh7 coculture model compared with the numbers of parasites containing empty vector (Fig. 1C). Next, we quantified the parasite levels by measuring the amount of genomic DNA; we compared the growth levels of wild-type and GRA15-KO parasites in THP-1 and Huh7 cells or in the coculture (Fig. 1D and E; see also Fig. S1F). The numbers of GRA15-KO parasites in IFN-γ-prestimulated THP-1 and Huh7 cells were similar to those seen with the wild-type parasites (Fig. 1D). In contrast, the numbers of GRA15-KO parasites were significantly lower than the numbers of wild-type parasites in cocultures of infected THP-1 and Huh7 cells in the presence of IFN-γ (Fig. 1E); this is consistent with data from luciferase assays. Collectively, these data suggest that GRA15 has an advantageous effect on T. gondii growth under these human cell line coculture conditions.

10.1128/mBio.01738-18.1 FIG S1 Generation of GRA15-deficient T. gondii by CRISPR/Cas9 genome editing. (A) Schematic of CRISPR/Cas9-mediated disruption of GRA15 by insertion of HXGPRT. Transfection of sg GRA15-1 and sg GRA15-2 together with the HXGPRT amplicon shown was used to disrupt the GRA15 coding region. The red bar indicates the sg GRA15-1 and sg GRA15-2 target region. (B) Quantitative RT-PCR analysis of GRA15 mRNA level in WT or GRA15-KO Pru T. gondii was performed, and the data was normalized to the mRNA expression levels of tubline in each samples. (C) THP-1 or Huh7 cells were infected with WT or GRA15-KO Pru T. gondii. The parasite number of indicated time points was measured by luciferase assay (D) THP-1 or Huh7 cells were infected with WT or GRA15-KO Pru T. gondii and then left untreated or treated with IFN-γ. The parasite survival rates were measured by luciferase assay. (E) Quantitative RT-PCR analysis of GRA15 mRNA level in GRA15-KO parasites stably expressing empty or GRA15 expression vectors was performed, and the data was normalized to the mRNA expression levels of tubulin in each samples. (F) The standard curve was established by the number ranging from 5 × 10^2^ to 5 × 10^6^ parasites and by qPCR cycle number of the SAG1 gene DNA. Indicated values represent means ± SD (three biological replicates per group from three independent experiments). (B, C, D, E, F). ***, *P* < 0.001; N.S., not significant (Student’s *t* test). Download FIG S1, PDF file, 0.1 MB. Copyright © 2018 Bando et al. 2018 Bando et al. This content is distributed under the terms of the Creative Commons Attribution 4.0 International license.

Next, we tested whether the proparasitic effect of GRA15 was present in a coculture of primary mouse macrophages and hepatocytes (Fig. 1F). In cocultures of primary mouse cells, the advantageous effect of GRA15 in IFN-γ-stimulated cells was not observed (Fig. 1F), indicating that the proparasitic effect of GRA15 might be absent in mice.

### IL-1 signaling in Huh7 cells downregulates the IFN-γ-induced anti-T. gondii response.

**FIG 2:**
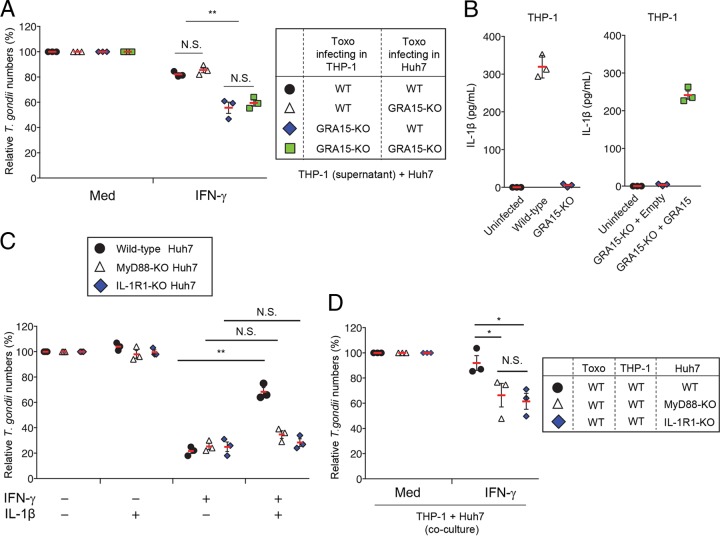
IL-1β production and signaling are important for the GRA15-dependent virulence mechanism. (A) THP-1 cells were infected with WT or GRA15-KO Pru T. gondii. The filtered culture supernatant was placed directly on top of the Huh7 cells, and the cells were newly infected with WT or GRA15-KO Pru T. gondii and then left untreated or treated with IFN-γ. The parasite survival rates were measured by luciferase assay. (B) THP-1 cells were left uninfected or infected with WT or GRA15-KO Pru T. gondii (left) or with GRA15-KO Pru T. gondii stably expressing GRA15 or empty vectors (right). Levels of IL-1β released into the culture supernatant were measured by ELISA. (C) WT, MyD88-KO, or IL-1R1-KO Huh7 cells were left untreated or treated with the indicated cytokines for 24 h and then infected with Pru T. gondii. The parasite survival rates were measured by luciferase assay. (D) THP-1 cells were infected with Pru T. gondii. The infected THP-1 cells were cocultured with WT, MyD88-KO, or IL-1R1-KO Huh7 cells and then left untreated or treated with IFN-γ. The parasite survival rates were measured by luciferase assay. Indicated values represent means ± SD (three biological replicates per group from three independent experiments) (panels A to D). **, *P* < 0.01; * *P* < 0.05; N.S., not significant (Student's *t* test).

To investigate which host factors are responsible for the GRA15-dependent proparasitic effect, we first compared levels of cell viability between wild-type and GRA15-KO T. gondii under the THP-1/Huh7 coculture conditions (Fig. S2A). Rates of cell death in coculture with wild-type or GRA15-KO parasites were comparable among the various time points tested (Fig. S2A), indicating that cell viability is not a factor for the GRA15-mediated proparasitic effect. Next, the supernatants from wild-type or GRA15-KO T. gondii-infected THP-1 cultures were collected, filtered to remove parasites or THP-1 cells, and subsequently added to Huh7 cells newly infected with wild-type or GRA15-KO parasites. The numbers of parasites in Huh7 cells were then assessed (Fig. 2A). We found that the presence of GRA15 in T. gondii parasites that infected the THP-1 cells, but not in those that infected the Huh7 cells, affected the GRA15-dependent increase in parasite numbers in the Huh7 cells (Fig. 2A). THP-1 cell infections with T. gondii show GRA15-dependent and strain-specific IL-1β production (23). We confirmed that IL-1β production occurred in THP-1 cells infected with wild-type or GRA15-KO parasites complemented with GRA15 but not in THP-1 cells infected with GRA15-KO parasites (Fig. 2B). On the other hand, IL-1β production did not occur in Huh7 cells infected with wild-type or GRA15-KO parasites (Fig. S2B). Because the results seen with the supernatant from the THP-1-infected cells explain the observed effect of GRA15 on the cells (Fig. 2A), we tested whether IL-1β is involved in parasite growth in Huh7 cells stimulated with IFN-γ (Fig. 2C). Stimulation with IFN-γ strongly suppressed the growth of the wild-type parasite in Huh7 cells (Fig. 2C). In contrast, the addition of IL-1β significantly impaired the IFN-γ-induced reduction in parasite numbers (Fig. 2C), whereas the effect of IL-1β was not observed when IFN-γ-pretreated Huh7 cells were subsequently infected with T. gondii and then stimulated with IL-1β (Fig. S2C). To directly test the effect of IL-1β signaling in Huh7 cells on downregulation of the IFN-γ-induced response, we utilized CRISPR/Cas9 genome editing to generate Huh7 cells lacking IL-1R1 or MyD88 (Fig. S2D and E), both of which are essential for the IL-1 receptor signaling pathway (39); we then examined the IL-1β-induced impairment of the IFN-γ-induced reduction in parasite numbers in the IL-1R1-KO or MyD88-KO Huh7 cells (Fig. 2C). Notably, IL-1β-induced impairment of the IFN-γ-mediated response against T. gondii was not observed in either IL-1R1-KO or MyD88-KO Huh7 cells (Fig. 2C). Furthermore, IL-1R1-KO or MyD88-KO Huh7 cells cocultured with the wild-type T. gondii-infected THP-1 cells contained significantly fewer parasites than the wild-type T. gondii-infected Huh7 cells (Fig. 2D). In addition, IL-1α, as well as IL-1β, had similar suppressive effects on the IFN-γ-induced reduction of T. gondii numbers in Huh7 cells (Fig. S2F). Collectively, these results suggest that IL-1 negatively regulates the IFN-γ-induced anti-T. gondii response in the Huh7 hepatocyte line.

10.1128/mBio.01738-18.2 FIG S2 Generation of MyD88- or IL-1R1-deficient Huh7 cells and caspase-1-, NLRP1- or NLRP3-deficient THP-1 cells by CRISPR/Cas9 genome editing. (A) Cell viability was measured by the LDH assay. THP-1 cells were infected with wild-type or GRA15-KO Pru T. gondii. The infected THP-1 cells were cocultured with Huh7 cells and then left untreated or treated with IFN-γ. The release of LDH of indicated time points was measured by the use of an LDH cytotoxicity colorimetric assay kit. Uninfected cells were used as a negative control, and Triton X-100-treated cells were used as a positive control. (B) Huh7 cells were left uninfected or infected with WT or GRA15-KO Pru T. gondii. IL-1β released into the culture supernatant was measured by ELISA. (C) •, Huh7 cells were left untreated or treated with the indicated cytokines for 24 h and then infected with Pru T. gondii; △, Huh7 cells were left untreated or treated with IFN-γ for 24 hours and then infected with Pru T. gondii with or without IL-1β. The parasite survival rate was measured by luciferase assay. (D and E) WT, MyD88-KO (D), or IL-1R1-KO (E) Huh7 cell lysates were detected by Western blotting. (F) Huh7 cells were left untreated or treated with the indicated cytokines for 24 h and then infected with Pru T. gondii. The parasite survival rates were measured by luciferase assay. (G to I) WT, CASP1-KO (G), NLRP1-KO (H), or NLRP3-KO (I) THP-1 cell lysates were detected by Western blotting. Each Western blot image is representative of three independent experiments (D, E, and G to I). Indicated values represent means ± SD (three biological replicates per group from three independent experiments) (A, B, C, and F). ***, *P* < 0.001; **, *P* < 0.01; N.S., not significant (Student’s *t* test). Download FIG S2, PDF file, 0.2 MB. Copyright © 2018 Bando et al. 2018 Bando et al. This content is distributed under the terms of the Creative Commons Attribution 4.0 International license.

### NLRP3 and caspase-1 are required for the proparasite effect of GRA15 in THP-1 cells.

**FIG 3:**
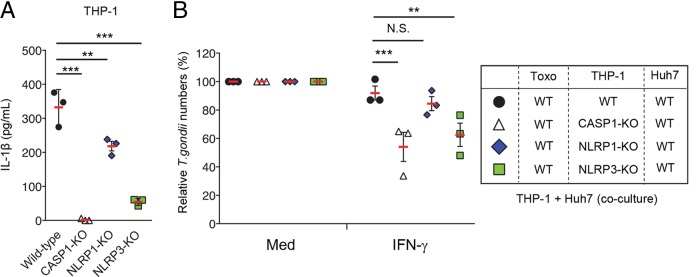
Caspase-1 and NLRP3 in THP-1 cells are involved in T. gondii-mediated IDO1 inhibition. (A and B) WT, CASP1-KO, NLRP1-KO, or NLRP3-KO THP-1 cells were left uninfected or infected with Pru T. gondii. (A) Levels of IL-1β released into the culture supernatant were measured by ELISA. (B) The infected THP-1 cells were cocultured with Huh7 cells and then left untreated or treated with IFN-γ. The parasite survival rates were measured by luciferase assay. Indicated values represent means ± SD (three biological replicates per group from three independent experiments) (panels A and B). ***, *P* < 0.001; **, *P* < 0.01; N.S., not significant (Student's *t* test).

The IL-1β produced in T. gondii-infected monocytes is dependent on the activities of caspase-1, NLRP1, and NLRP3 (23, 40). Therefore, to test whether caspase-1, NLRP1, and NLRP3 are important for the proparasitic effect of GRA15, we generated caspase-1-deficient (CASP1-KO), NLRP1-deficient (NLRP1-KO), and NLRP3-deficient (NLRP3-KO) THP-1 cells by using CRISPR/Cas9 genome editing (Fig. S2G to I). We next tested IL-1β production in infections with T. gondii (Fig. 3A). THP-1 cells that lacked caspase-1 or NLRP3 were severely defective in IL-1β production in the parasite infections (Fig. 3A). In contrast, NLRP1 deficiency in THP-1 cells resulted in a minimal defect in IL-1β production (Fig. 3A). Then, to assess whether IL-1β production during T. gondii infection in THP-1 cells was essential for suppressing the IFN-γ-induced antiparasite response in our coculture model, we examined whether IFN-γ-induced parasite reduction requires caspase-1, NLRP1, or NLRP3. The numbers of parasites in the wild-type parasite-infected CASP1-KO and wild-type parasite-infected NLRP3-KO THP-1 cells, but not wild-type parasite-infected NLRP1-KO THP-1 cells, cocultured with wild-type Huh7 cells were lower than those seen with the wild-type parasite-infected parental THP-1 cells (Fig. 3B), indicating that IL-1β production through activation of the NLRP3 inflammasome, but not the NLRP1 inflammasome, in THP-1 cells is important for GRA15-mediated suppression of the IFN-γ-mediated anti-T. gondii response.

### GRA15 indirectly downregulates the IDO1-induced anti-T. gondii cell-autonomous immune response.

**FIG 4:**
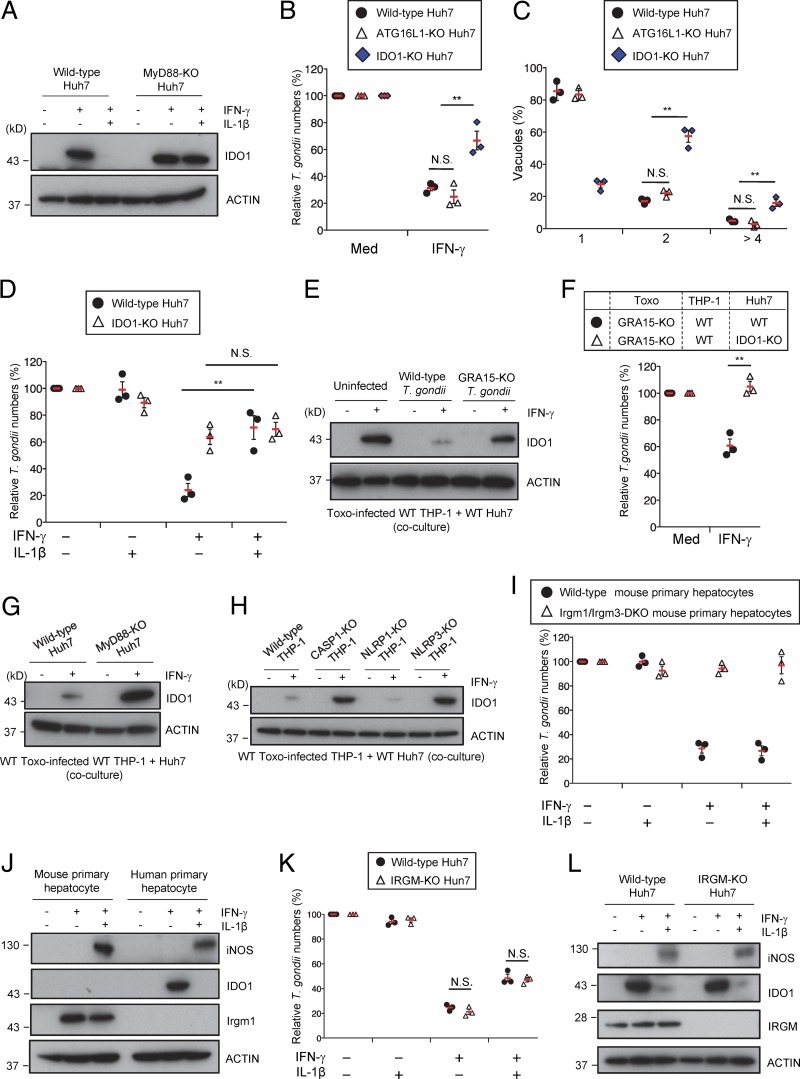
IL-1β production and signaling are essential for GRA15-mediated IDO1 inhibition. (A) WT or MyD88-KO Huh7 cells were left untreated or treated with the indicated cytokines. Expression of IDO1 in the cell lysates was detected by Western blotting. (B and C) WT, Atg16L1-KO, or IDO1-KO Huh7 cells were left untreated or treated with IFN-γ for 24 h and then infected with Pru T. gondii. (B) The parasite survival rates were measured by luciferase assay. (C) The parasite number per vacuole after 24 h postinfection was measured by indirect immunofluorescent assay (IFA). (D) WT or IDO1-KO Huh7 cells were left untreated or treated with the indicated cytokines for 24 h and then infected with Pru T. gondii. The parasite survival rates were measured by luciferase assay. (E) THP-1 cells were infected with WT or GRA15-KO Pru T. gondii. The infected THP-1 cells were cocultured with Huh7 cells and then left untreated or treated with IFN-γ. Expression of IDO1 in the cell lysates was detected by Western blotting. (F) THP-1 cells were infected with GRA15-KO Pru T. gondii. The infected THP-1 cells were cocultured with WT or IDO1-KO Huh7 cells and then left untreated or treated with IFN-γ. The parasite survival rates were measured by luciferase assay. (G) THP-1 cells were infected with Pru T. gondii. The infected THP-1 cells were cocultured with WT or MyD88-KO Huh7 cells and then left untreated or treated with IFN-γ. Expression of IDO1 in cell lysates was detected by Western blotting. (H) WT, CASP1-KO, NLRP1-KO, or NLRP3-KO THP-1 cells were left uninfected or infected with Pru T. gondii. The infected THP-1 cells were cocultured with Huh7 cells and then left untreated or treated with IFN-γ. Expression of IDO1 in the cell lysates was detected by Western blotting. (I) WT or Irgm1/3 double-knockout (DKO) primary mouse hepatocytes were left untreated or treated with the indicated cytokines for 24 h and then infected with Pru T. gondii. The parasite survival rates were measured by luciferase assay. (J) Primary human hepatocyte or primary mouse hepatocytes were left untreated or treated with the indicated cytokines. Expression of indicated proteins in cell lysates was detected by Western blotting. (K) WT or IRGM-KO Huh7 cells were left untreated or treated with the indicated cytokines for 24 h and then infected with wild-type Pru T. gondii. The parasite survival rates were measured by luciferase assay. (L) WT or IRGM-KO Huh7 cells were left untreated or treated with the indicated cytokines. Expression of indicated proteins in cell lysates was detected by Western blotting. Each Western blot image is representative of three independent experiments (A, E, G, H, J, and L). Indicated values represent means ± SD (three biological replicates per group from three independent experiments) (B to D, F, I, and K). **, *P* < 0.01; N.S., not significant (Student's *t* test).

IFN-γ-induced tryptophan degradation by IDO plays a central role in the IFN-γ-induced anti-T. gondii response in humans (34). Interestingly, we found that IL-1β costimulation severely reduced IDO1 protein levels in a MyD88-dependent fashion (Fig. 4A). Because the reported results of previous studies in this area were based on the use of the 1-MT IDO inhibitor, there is no genetic evidence for the involvement of IDO in the anti-T. gondii response. Furthermore, ATG16L1, an autophagy protein, has recently been reported to participate in the IFN-γ-induced antiparasite response in a HeLa cervical carcinoma cell line (41). Therefore, we generated IDO1-deficient (IDO1-KO) and ATG16L1-deficient (ATG16L1-KO) Huh7 cells by using CRISPR/Cas9 genome editing to determine whether IDO or ATG16L1 or both are important for the IFN-γ-induced anti-T. gondii response in Huh7 cells (Fig. S3A and B). Because kynurenine is a tryptophan metabolite of IDO (42), we measured the kynurenine concentration in IDO1-KO Huh7 cells (Fig. S3C). While the kynurenine concentration increased upon IFN-γ treatment of wild-type Huh7 cells, such an increase was not observed in IDO1-KO cells (Fig. S3C). We then assessed whether an IFN-γ-induced reduction in T. gondii growth and parasite numbers in vacuoles had occurred in the wild-type, ATG16L1-KO, or IDO1-KO cells (Fig. 4B and C). The wild-type and ATG16L1-KO cells showed similar reductions in parasite numbers in response to IFN-γ. In sharp contrast, impaired IFN-γ-induced reductions in parasite numbers, as well as increased parasite growth in each parasitophorous vacuole, were observed in IDO1-KO cells (Fig. 4B and C), suggesting that IDO1, but not ATG16L1, plays an important role in the IFN-γ-induced anti-T. gondii cell-autonomous response. Comparing the levels of IL-1β-induced impairment of the IFN-γ-dependent response between wild-type and IDO1-KO Huh7 cells, the effect of IL-1β was found to have been completely abolished in IDO1-KO cells (Fig. 4D). Consequently, we tested whether the parasite number reduction caused by GRA15 deficiency involves IDO1 (Fig. 4E). We compared IDO1 protein levels in uninfected and wild-type or GRA15-KO parasite-infected THP-1/Huh7 cocultures (Fig. 4E). Although the wild-type parasite-infected condition resulted in low IDO1 protein levels, the levels of IDO1 protein in the GRA15-KO parasite-infected condition were higher than those seen under the wild-type parasite-infected conditions (Fig. 4E). Moreover, the IFN-γ-induced reduction in the numbers of GRA15-KO parasites was abolished when Huh7 cells lacked IDO1 (Fig. 4F), suggesting that the reduction in parasite numbers related to GRA15 deficiency was caused by increased IDO1 protein levels. In addition, we consistently found that IDO1 protein levels in cocultures of wild-type parasite-infected wild-type THP-1 and MyD88-KO Huh7 cells were higher than those seen with wild-type Huh7 cells (Fig. 4G). Furthermore, IDO1 protein levels in cocultures of CASP1-KO or NLRP3-KO, but not NLRP1-KO, THP-1 cells and wild-type Huh7 cells were higher than those seen with the wild-type THP-1 cells (Fig. 4H). Taken together, these data showed that infection of THP-1 cells with GRA15-intact T. gondii produced IL-1β in a manner dependent on NLRP3 and caspase-1; this led to an indirect reduction in IDO1 proteins, thereby supporting parasite growth in human cells.

10.1128/mBio.01738-18.3 FIG S3 Generation of IDO1-, ATG16L1-, or IRGM-deficient Huh7 cells by CRISPR/Cas9 genome editing. (A) WT or IDO1-KO Huh7 cells were left untreated or treated with IFN-γ. Expression of IDO1 in the cell lysates was detected by Western blotting. (B) WT or ATG16L1-KO Huh7 cell lysates were detected by Western blotting. (C) The concentration of kynurenine in the culture supernatant was measured. (D) WT or IRGM-KO Huh7 cell lysates were detected by Western blotting. Each Western blot image is representative of three independent experiments (A, B, and D). Indicated values represent means ± SD (three biological replicates per group from three independent experiments) (C). Download FIG S3, PDF file, 0.1 MB. Copyright © 2018 Bando et al. 2018 Bando et al. This content is distributed under the terms of the Creative Commons Attribution 4.0 International license.

The proparasitic effect of GRA15 in the presence of IFN-γ was not observed under the mouse macrophage/hepatocyte coculture conditions (Fig. 1F). Next, we tested whether the proparasitic effect of IL-1β was present in IFN-γ-activated mouse hepatocytes, in terms of IDO1 (Fig. 4I and J). Mouse hepatocytes also showed strong IFN-γ-mediated suppression of T. gondii growth (Fig. 4I). However, in sharp contrast to the observations in human cells, IL-1β stimulation in mouse hepatocytes could not reverse the IFN-γ-induced reduction of T. gondii levels (Fig. 4I). Furthermore, mouse hepatocytes lacking IFN-inducible GTPases, such as Irgm1 and Irgm3 (well-known anti-T. gondii factors in mice) (43), were completely defective with respect to IFN-γ-induced parasite number reduction (Fig. 4I). Comparing the IDO1 protein levels in human and mouse hepatocytes, IDO1 proteins were detected in human cells but not in mouse cells (Fig. 4J). In contrast, Irgm1 proteins were detected only in mouse cells, and not in human cells, in response to IFN-γ (Fig. 4J); notably, humans do not express Irgm1 but possess IRGM, a single human homolog of IRG whose significance remains unclear with respect to the anti-T. gondii human response (44). To directly examine the role of IRGM in Huh7 cells, we generated IRGM-KO Huh7 cells by CRISPR/Cas9 genome editing and tested for the ability to mount an IFN-γ-induced anti-T. gondii response (Fig. 4K and L; see also Fig. S3D). IFN-γ-prestimulated IRGM-KO cells reduced T. gondii numbers in a manner similar to that seen with the wild-type cells (Fig. 4K). Furthermore, the IL-1β-mediated increases of parasite numbers and concomitant reductions of IDO1 protein levels in IFN-γ-stimulated IRGM-KO Huh7 cells were similar to those observed in wild-type cells (Fig. 4K and L). Taken together, these results indicate that the IL-1-mediated proparasitic effect is not present in mouse hepatocytes, where IFN-inducible GTPases such as Irgm1 and Irgm3 play major roles in the anti-T. gondii system. In addition, human IRGM is not involved in the IFN-γ-induced anti-T. gondii response and the IL-1-mediated proparasitic effect in human cells.

### iNOS is essential for the GRA15-dependent reduction in IDO1 protein levels.

**FIG 5:**
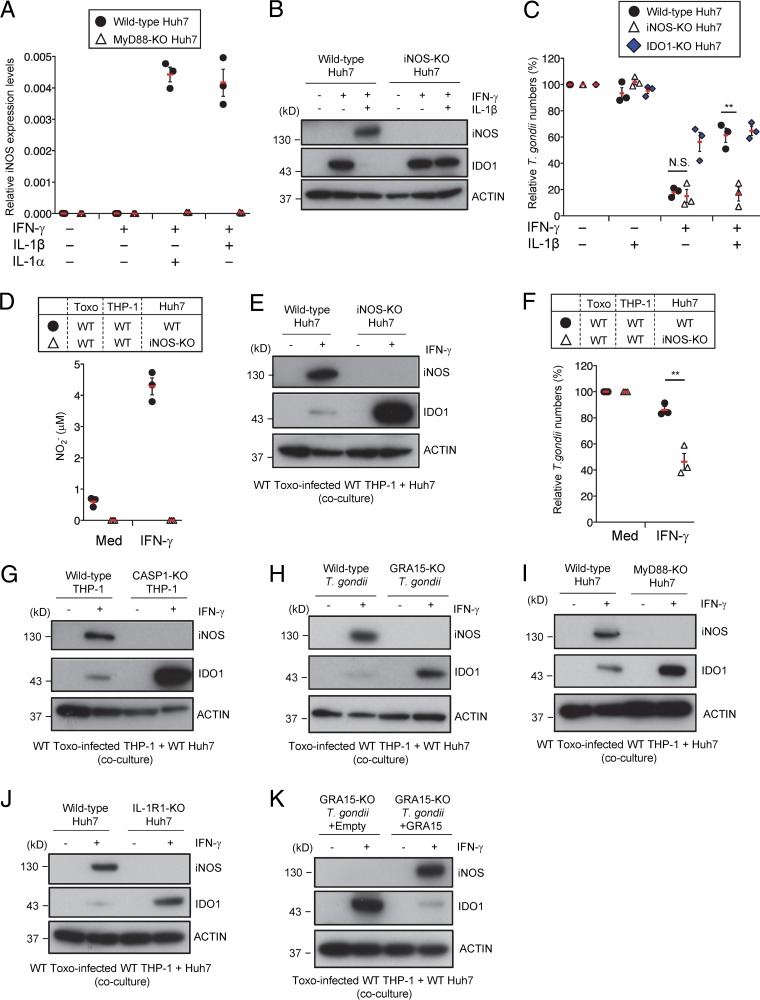
iNOS expression in Huh7 cells is required for GRA15-mediated IDO1 inhibition. (A) Quantitative RT-PCR analysis of iNOS mRNA level in WT or MyD88-KO Huh7 cells that were left untreated or treated with the indicated cytokines was performed. (B) WT or iNOS-KO Huh7 cells were left untreated or treated with the indicated cytokines. Expression of indicated proteins in the cell lysates was detected by Western blotting. (C) WT, iNOS-KO, or IDO1-KO Huh7 cells were left untreated or treated with the indicated cytokines for 24 h and then infected with Pru T. gondii. The parasite survival rates were measured by luciferase assay. (D to F) THP-1 cells were infected with Pru T. gondii. The infected THP-1 cells were cocultured with WT or iNOS-KO Huh7 cells and then left untreated or treated with IFN-γ. (D) Levels of NO_2_ released into the culture supernatant were measured by ELISA. (E) Expression of indicated proteins in the cell lysates was detected by Western blotting. (F) The parasite survival rates were measured by luciferase assay. (G to K) WT or CASP1-KO (G) THP-1 cells were infected with WT or GRA15-KO Pru T. gondii (H) or with GRA15-KO parasites stably expressing empty or GRA15 expression vectors (K). The infected THP-1 cells were cocultured with WT, MyD88-KO (I), or IL-1R1-KO (J) Huh7 cells and then left untreated or treated with IFN-γ. Expression of indicated proteins in the cell lysates was detected by Western blotting. Each Western blot image is representative of three independent experiments (B, E, and G to K). Indicated values represent means ± SD (three biological replicates per group from three independent experiments) (A, C, D, and F). **, *P* < 0.01; N.S., not significant (Student's *t* test).

We attempted to gain further insight into the mechanism by which IL-1β-mediated suppression of IDO1 protein levels is dependent on MyD88. NO is known to inhibit IDO activity in macrophages (45). Because iNOS is important for IFN-γ-mediated NO production (46), we examined iNOS mRNA expression in Huh7 cells (Fig. 5A). Stimulation with IL-1α or IL-1β in addition to IFN-γ strongly induced iNOS mRNA expression and NO production in a MyD88-dependent manner (Fig. 5A; see also Fig. S4A). Furthermore, THP-1 cells did not express iNOS protein and produce NO in response to IFN-γ or/and IL-1β stimulation (Fig. S4B and C). Therefore, to assess the role of iNOS in Huh7 cells, we generated iNOS-deficient (iNOS-KO) Huh7 cells by CRISPR/Cas9 genome editing (Fig. S4B). The iNOS-KO Huh7 cells lacked NO production in response to IL-1β/IFN-γ (Fig. S4D and E). Furthermore, IL-1β-induced reduction of IDO1 protein levels and impaired IFN-γ-dependent parasite reduction were not observed in iNOS-KO cells (Fig. 5B and C), suggesting that IL-1β might stimulate iNOS expression and thereby cause IDO1 inhibition, serving as the GRA15-dependent virulence mechanism. We tested this possibility by using the parasite-infected THP-1/Huh7 coculture model (Fig. 5D and E). iNOS-KO Huh7 cells cocultured with wild-type parasite-infected wild-type THP-1 cells exhibited a complete loss of NO production but showed no reduction in IDO1 protein levels (Fig. 5D and E). Additionally, the IFN-γ-mediated reduction in numbers of parasites was enhanced in cocultures of iNOS-KO Huh7 cells, in comparison with wild-type cells (Fig. 5F). When CASP1-KO THP-1 cells, IL-1R1-KO Huh7 cells, MyD88-KO Huh7 cells, or GRA15-KO T. gondii were used in the coculture model, iNOS protein expression and NO production were defective (Fig. 5G to J; see also Fig. S4F), suggesting that IL-1β-induced NO is not important for the anti-T. gondii response in Huh7 cells. Moreover, complementation of GRA15 in GRA15-KO parasites restored iNOS expression and reduction of IDO1 protein levels in the THP-1/Huh7 coculture model (Fig. 5K). Taken together, these data indicate that iNOS is critical for the GRA15-dependent virulence mechanism in the THP-1/Huh7 coculture model.

10.1128/mBio.01738-18.4 FIG S4 MyD88- and iNOS-dependent NO production in response to IL-1β and IFN-γ in Huh7 cells. (A) WT or MyD88-KO Huh7 cells were left untreated or treated with the indicated cytokines. Levels of NO_2_ released into the culture supernatant were measured by ELISA. (B and C) THP-1 cells alone were stimulated with indicated cytokines for 24 h and then uninfected or infected with Pru T. gondii. The infected THP-1/Huh7 coculture condition was used for the positive control. (B) The levels of expression of iNOS in the cell lysates were detected by Western blotting. (C) Levels of NO_2_ released into the culture supernatant were measured by ELISA. (D and E) WT or iNOS-KO Huh7 cells were left untreated or treated with the indicated cytokines. (D) iNOS expression in the cell lysates was detected by Western blotting. (E) Levels of NO_2_ released into the culture supernatant were measured by ELISA. (F) WT or CASP1-KO THP-1 cells were infected with WT or GRA15-KO T. gondii. The infected THP-1 cells were cocultured with WT, MyD88-KO, or IL-1R1-KO Huh7 cells and then left untreated or treated with IFN-γ. Levels of NO_2_ released into the culture supernatant were measured by ELISA. Western blot images are representative of three independent experiments (B and D). Indicated values represent means ± SD (three biological replicates per group from three independent experiments) (A, C, E, and F). Download FIG S4, PDF file, 0.2 MB. Copyright © 2018 Bando et al. 2018 Bando et al. This content is distributed under the terms of the Creative Commons Attribution 4.0 International license.

### Blockade of NO production inhibits T. gondii growth in THP-1/Huh7 coculture condition.

**FIG 6:**
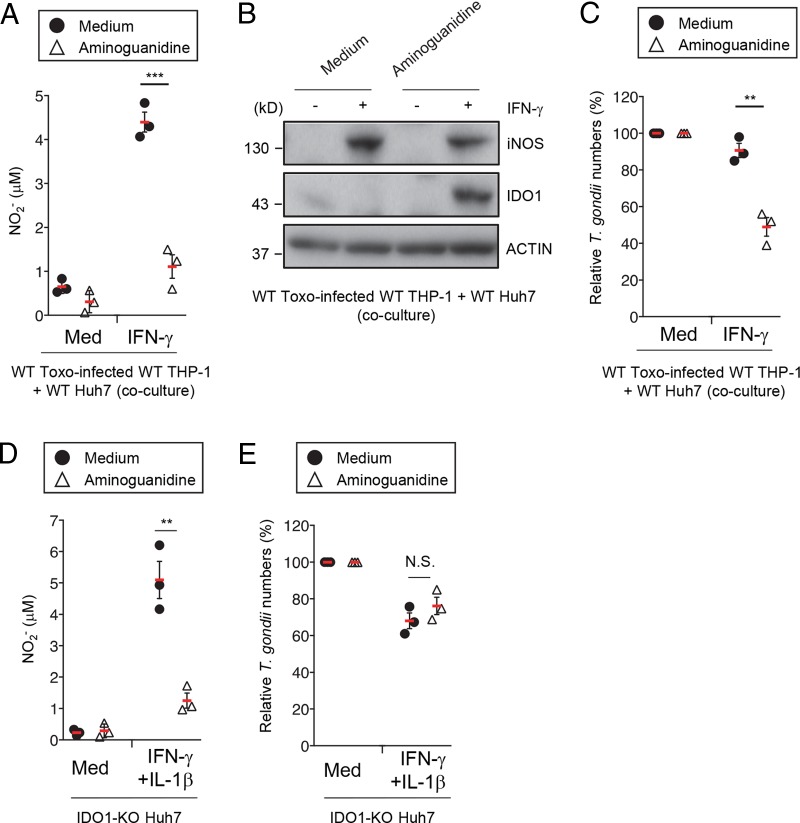
iNOS inhibitor prevents GRA15-dependent production of NO and reduction of IDO1 proteins. (A to C) THP-1 cells were infected with Pru T. gondii. The infected THP-1 cells were cocultured with Huh7 cells and then left untreated or treated with IFN-γ and/or aminoguanidine. (A) The levels of NO_2_ released into the culture supernatant were measured by ELISA. (B) Expression of indicated proteins in the cell lysates was detected by Western blotting. (C) The parasite survival rates were measured by luciferase assay. (D and E) WT or IDO1-KO Huh7 cells were left untreated or treated with the indicated cytokines and/or aminoguanidine and infected with Pru T. gondii. (D) Levels of NO_2_ released into the culture supernatant were measured by ELISA. (E) The parasite survival rates were measured by luciferase assay. The Western blot image is representative of three independent experiments (B). Indicated values represent means ± SD (three biological replicates per group from three independent experiments) (A, C, D, and E). ***, *P* < 0.001; **, *P* < 0.01; N.S., not significant (Student's *t* test).

The essential role of iNOS in the proparasitic effect of GRA15 prompted us to examine whether this step can be targeted by a specific inhibitor. To test whether NO produced by iNOS is critical for the GRA15-mediated reduction in IDO1 levels, we used aminoguanidine, an inhibitor of NO synthase, and calculated the numbers of parasites (47). Addition of aminoguanidine to the coculture reduced the NO concentration and, conversely, increased IDO1 protein levels; in contrast, the iNOS level remained unchanged between the control and aminoguanidine-treated cocultures (Fig. 6A and B), indicating that aminoguanidine inhibits NO production downstream of iNOS and restores IDO1 protein levels. Consequently, the number of parasites in the coculture system was significantly reduced in the presence of aminoguanidine (Fig. 6C). Although aminoguanidine treatment of the IDO1-KO Huh7 cells also inhibited NO production, the IFN-γ-induced reduction in parasite numbers was unchanged between the aminoguanidine-treated and untreated cells (Fig. 6D and E), suggesting that the NO levels might be insufficient for the suppression of T. gondii growth in Huh7 cells or that NO might have an anti-T. gondii effect in Huh7 cells. Taken together, these results demonstrate that inhibition of NO production by aminoguanidine may ameliorate the proparasitic effect of GRA15 and the corresponding IL-1-mediated virulence mechanism.

### GRA15-dependent IDO1 reduction by iNOS-mediated NO in coculture of primary human monocytes and hepatocytes.

**FIG 7:**
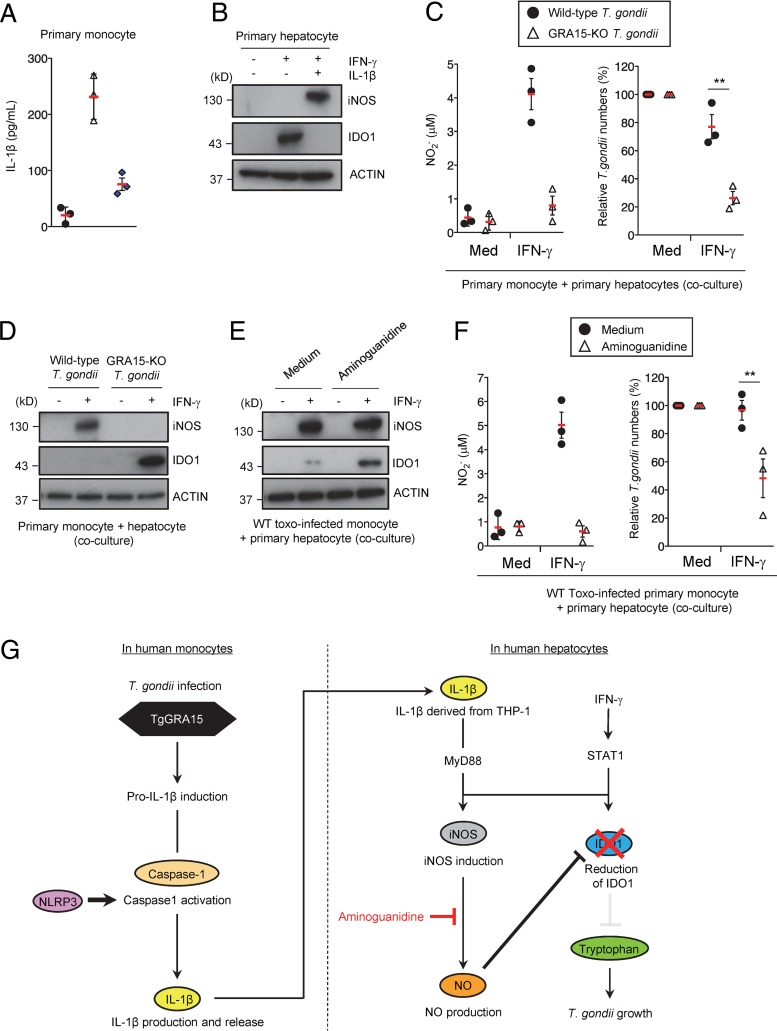
The GRA15-dependent virulence mechanism is operative under primary human monocyte/hepatocyte coculture conditions. (A) Primary human monocytes were left uninfected or infected with WT or GRA15-KO Pru T. gondii. Levels of IL-1β released into the culture supernatant were measured by ELISA. (B) Primary human hepatocytes were left untreated or treated with the indicated cytokines. Expression of indicated proteins in the cell lysates was detected by Western blotting. (C and D) Primary human monocytes were infected with WT or GRA15-KO Pru T. gondii. The infected monocytes were cocultured with primary human hepatocytes and then left untreated or treated with IFN-γ. (C, left panel) Levels of NO_2_ released into the culture supernatant were measured by ELISA. (C, right panel) The parasite survival rates were measured by luciferase assay. (D) Expression of the indicated proteins in the cell lysates was detected by Western blotting. (E and F) Primary human monocytes were infected with Pru T. gondii. The infected monocytes were cocultured with primary human hepatocytes and then left untreated or treated with IFN-γ and/or aminoguanidine. (E) Expression of indicated proteins in the cell lysates was detected by Western blotting. (F, left panel) NO_2_ released into the culture supernatant was measured by ELISA. (F, right panel) The parasite survival rates were measured by luciferase assay. (G) GRA15-mediated indirect virulence program targeting IDO1 in the coculture model. T. gondii secreted effector GRA15 in the infected monocytes, which resulted in NLRP3-dependent IL-1β production. IL-1β from infected monocytes and IFN-γ induce the expression of iNOS and NO production, leading to indirect reduction of levels of IDO1 proteins in hepatocytes and allowing T. gondii growth. Each Western blot image is representative of three independent experiments (B, D, and E). Indicated values represent means ± SD (three biological replicates per group from three independent experiments) (A, C, and F). **, *P* < 0.01; N.S., not significant (Student's *t* test).

Finally, we tested whether the T. gondii GRA15-mediated virulence program is observed in primary human cells. Wild-type T. gondii infection in human _CD14_^+^ monocytes from peripheral blood caused IL-1β production, whereas infection by GRA15-KO parasites did not (Fig. 7A). Next, responses to IFN-γ and IL-1β, in terms of IDO1 and iNOS expression, were examined in primary hepatocytes (Fig. 7B). Levels of IDO1 proteins were severely reduced in primary hepatocytes (Fig. 7B), suggesting that primary monocytes and hepatocytes behaved in a manner similar to that shown by THP-1 and Huh7 cells. Then, we examined parasite growth in cocultures of T. gondii-infected primary monocytes with primary hepatocytes (Fig. 7C). The growth of GRA15-deficient T. gondii in the cocultures was much lower than the growth of wild-type parasites (Fig. 7C). Furthermore, the levels of NO concentration and iNOS expression in cocultures containing GRA15-deficient parasites were lower than those in cocultures containing wild-type parasites (Fig. 7C and D). Conversely, the level of IDO1 protein was higher in cocultures containing GRA15-deficient parasites than in cocultures containing wild-type parasites (Fig. 7D), indicating that the presence of GRA15 is advantageous for T. gondii in primary human cells. Next, we asked whether pharmacological blockade of NO production inhibits the growth of GRA15-intact wild-type T. gondii in cocultures of primary monocytes and hepatocytes (Fig. 7E and F). Aminoguanidine treatment in the coculture resulted in increased IDO1 protein levels and reduced NO concentrations, although iNOS protein levels were unchanged (Fig. 7E). Furthermore, wild-type T. gondii growth was significantly reduced in the presence of aminoguanidine (Fig. 7F). Taken together, these results indicate that the IFN-γ-induced anti-T. gondii human cell-autonomous response requires IDO1, which is inhibited by the GRA15 parasite effector in a manner that utilizes host NLRP3 inflammasome and iNOS, in cocultures of T. gondii-infected human cells (Fig. 7G).

## DISCUSSION

The present study has shown that iNOS, a well-known, critical anti-T. gondii host factor in mice (35), acts as a “pro-T. gondii” host factor in humans. iNOS participates in the GRA15-dependent virulence mechanism to suppress the IFN-γ-induced IDO1-mediated antiparasite human response.

There are three major pathways by which iNOS downregulates IDO1 expression levels. NO produced by iNOS negatively regulates IFN-γ-stimulated transcription of IDO mRNA (48). In addition, NO directly binds to IDO and inhibits its enzymatic activity (49). Finally, NO induces degradation of IDO in a proteasome-dependent fashion (50). Notably, the molecular mechanisms by which iNOS-generated NO negatively regulates IDO1 expression levels have been well studied. In the current study, we demonstrated that T. gondii exploits the iNOS (NO)-dependent downregulation of IDO1 by the secreting effector GRA15 to proliferate efficiently in a variety of human cell types. We found that GRA15-intact T. gondii infection indirectly reduces IDO1 protein levels via iNOS expressed in certain secondary cells, which is responsive to IL-1β derived from infected THP-1 cells or primary monocytes. In mice, genetic ablation of IDO1 has been shown to inhibit T. gondii proliferation *in vivo* (51), suggesting that IDO1 promotes T. gondii growth in mice. Conversely, pharmacological blockade of IDO by 1-MT in mice leads to defective parasite clearance *in vivo* (52). Thus, controversy exists regarding the anti-T. gondii role of IDO1 in mouse models. In contrast, iNOS is well established as a critical anti-T. gondii factor in mice (35). IFN-γ-activated mouse macrophages show neither IDO activity nor tryptophan degradation but can inhibit T. gondii growth by using the NO produced via iNOS (35). However, this study and another (28) have both shown that NO may not play a significant role in human hepatocytes, monocytes, or macrophages; in contrast, a different study showed that NO can inhibit T. gondii growth in human astrocytes (53). Thus, the role of NO and iNOS in the human response against T. gondii has been unclear. Although nitrite levels in murine cells can reach concentrations greater than 100 μM (54), those in human cells remain less than 10 μM, which might be too low for parasite growth inhibition. Given that IFN-γ stimulates IDO1 expression in many human cell types (33, 34), humans may have evolutionarily adopted IDO1 for primary anti-T. gondii cell-autonomous immunity in hepatocytes; mice may have preferentially selected iNOS and IFN-γ-inducible GTPases for this purpose. Indeed, we failed to observe IDO1 expression in IFN-γ-stimulated mouse hepatocytes. Furthermore, the absence of IFN-γ-inducible GTPases in mouse hepatocytes completely abolished the IFN-γ-induced reduction of T. gondii numbers. Thus, such species-specific differences regarding dependence on IDO1 and IFN-γ-inducible GTPases in antiparasitic responses in humans and mice, respectively, may make the role of iNOS as a host-derived covirulence factor for GRA15 difficult to clearly elucidate in the mouse model (21, 22). The role played by GRA15 in suppressing IFN-γ-induced anti-T. gondii cell-autonomous immunity is evident only when GRA15-intact T. gondii parasites are used to infect normal monocytes that are then cocultured with normal hepatocytes. Because T. gondii preferentially infects _CD11b_^+^ cells (e.g., monocytes) and spreads throughout various tissues from the primary sites of infection *in vivo* (37), GRA15 might play a role in parasite proliferation in human liver tissue, where newly arrived monocytes infected with GRA15-intact T. gondii might produce IL-1β and inhibit IDO1 expression in an iNOS-dependent fashion.

Our current study has shown that GRA15, a protein in type II T. gondii, promotes parasite survival in IFN-γ-stimulated human cells. Of note, type II T. gondii is the most prevalent cause of both human congenital and acquired toxoplasmosis in North America and Europe (55–57). It is possible that type II GRA15-dependent IL-1β production, which suppresses the IFN-γ-induced IDO1-dependent immune response via iNOS, might be involved in the pathogenesis of human toxoplasmosis. Of note, treatment with an inhibitor of NO production reduced T. gondii numbers to levels below those seen under both THP-1/Huh7 and primary monocyte/hepatocyte coculture conditions, suggesting that pharmacological blockade of NO production might ameliorate human toxoplasmosis. Furthermore, a previous study also reported that costimulation with IL-1β and IFN-γ inhibited IDO activity mediated by NO in a Staphylococcus aureus-infected human cell line, thereby resulting in an impaired IFN-γ-mediated reduction in bacterial numbers (50). Thus, IL-1β might have a pathogenic function in the suppression of the IFN-γ-mediated, IDO1-dependent, cell-autonomous response to T. gondii and to various other human intracellular pathogens.

In summary, we have shown that T. gondii can suppress the IFN-γ-induced antiparasitic response by indirectly targeting IDO1 in hepatocytes cocultured with monocytes. By investigating differences between human and mouse immune responses, unidentified virulence mechanisms associated with known or unknown T. gondii effectors may be discovered in the future. Additionally, pharmacological blockade of NO production could offer a novel therapeutic strategy for treating human toxoplasmosis.

## MATERIALS AND METHODS

### Cells and parasites.

C57BL/6 mice (6 weeks of age) were obtained from SLC. Irgm1/Irgm3-deficient mice were maintained under specific-pathogen-free conditions. All animal experiments were conducted with the approval of the Animal Research Committee of the Research Institute for Microbial Diseases in Osaka University. T. gondii parasites were maintained in Vero cells in RPMI medium (Nacalai Tesque) supplemented with 2% heat-inactivated fetal bovine serum (FBS) (JRH Bioscience), 100 U/ml penicillin, and 0.1 mg/ml streptomycin (Nacalai Tesque), as previously described (20). Huh7 cells and THP-1 cells were maintained in RPMI medium containing 10% heat-inactivated FBS, 100 U/ml penicillin, and 0.1 mg/ml streptomycin. Cryopreserved primary human monocytes (c-12909) derived from three different individuals were obtained from TaKaRa and were maintained in RPMI medium containing 10% heat-inactivated FBS, 100 U/ml penicillin, and 0.1 mg/ml streptomycin. Cryopreserved primary human hepatocytes (HUCPQ) derived from three different individuals were obtained from Lonza and were thawed in single-human thawing medium (Lonza). Primary human hepatocytes were maintained in Williams’ E medium (Thermo Fisher Scientific) containing 10% heat-inactivated FBS, 100 U/ml penicillin, 0.1 mg/ml streptomycin, 2 mM l-glutamine (Sigma), and 10 mg/ml insulin (Roche). Bone-marrow-derived macrophages (BMDMs) were differentiated in RPMI medium containing 10% heat-inactivated FBS, 100 U/ml penicillin, 0.1 mg/ml streptomycin, and 10% L929 cell (ATCC) supernatant for 6 days. To prepare primary hepatocytes, 6-week-old male mice were anesthetized and their livers were isolated. Primary hepatocytes were prepared using liver digestion medium (Thermo Fisher Scientific) as previously described (58). Primary hepatocytes were maintained in Williams’ E medium (Life Technologies) containing 1× GlutaMAX-I (Life Technologies), 10% heat-inactivated FBS, 100 U/ml penicillin, 0.1 mg/ml streptomycin, 10 ng/ml epidermal growth factor (EGF) (Sigma), 1× GlutaMAX (Thermo Fisher Scientific), 2 mM l-glutamine (Sigma), and 10 mg/ml insulin (Roche).

### Reagents.

Antibodies against IDO1 (13268-1-AP), NLRP1 (12256-1-AP), NLRP3 (19771-1-AP), and caspase-1 (22915-1-AP) were obtained from Proteintech. Antibodies against Irgm1 (LRG47; sc-11075), IL-1R1 (H-8; sc-393998), and iNOS (NOS2; sc-7271) were obtained from Santa Cruz Biotechnology. Anti-IRGM antibody (ABC338) was obtained from Merck Millipore. Anti-β-actin antibody (A1978) was obtained from Sigma. Anti-MyD88 antibody (2127) and anti-ATG16L1 antibody (PM040) were obtained from ProSci and MBL, respectively. Antibodies against GAP45 were described previously (59). Recombinant human and mouse IFN-γ, IL-1α, and IL-1β were obtained from Peprotech. Aminoguanidine hydrochloride (396494) was obtained from Sigma. Purified anti-human IL-1β (511601) and biotin anti-human IL-1β (511703) were obtained from BioLegend.

### Plasmid construction for generation of human cell lines.

All genomically deficient cell lines were generated with a px330 plasmid CRISPR/Cas9 system. The insertion fragments of IDO1, iNOS, IL-1R1, IRGM, CASP1, NLRP1, NLRP3, MyD88, and ATG16L1 genomic DNA (gRNA) were generated by annealing primers. All the primers used in this study are listed in Table S1 in the supplemental material. These insertion fragments were inserted into the BbsI site of the cloning vector containing the U6 promoter to generate gRNA-expressing plasmids pIDO1_gRNA1, pIDO1_gRNA2, piNOS_gRNA1, piNOS_gRNA2, pIL-1R1_gRNA1, pIL-1R1_gRNA2, pIRGM_gRNA1, pIRGM_gRNA2, pCASP1_gRNA1, pCASP1_gRNA2, pNLRP1_gRNA1, pNLRP1_gRNA2, pNLRP3_gRNA1, pNLRP3_gRNA2, pMyD88_gRNA1, pMyD88_gRNA2, pATG16L1_gRNA1, and pATG16L1_gRNA2. The insertion fragment was cut out by XhoI and SalI from the pIDO1_gRNA2, piNOS_gRNA2, pIL-1R1_gRNA2, pIRGM_gRNA2, pCASP1_gRNA2, pNLRP1_gRNA2, pNLRP3_gRNA2, pMyD88_gRNA2, and pATG16L1_gRNA2 vector and ligated into the XhoI site of the pIDO1_gRNA1, piNOS_gRNA1, pIL-1R1_gRNA1, pIRGM_gRNA1, pCASP1_gRNA1, pNLRP1_gRNA1, pNLRP3_gRNA1, pMyD88_gRNA1, and pATG16L1_gRNA1 vector to generate plasmids pIDO1_gRNA1/2, piNOS_gRNA1/2, pIL-1R1_gRNA1/2, pIRGM_gRNA1/2, pCASP1_gRNA1/2, pNLRP1_gRNA1/2, pNLRP3_gRNA1/2, pMyD88_gRNA1/2, and pATG16L1_gRNA1/2. The insertion fragment was cut out by KpnI and MluI from pIDO1_gRNA1/2, piNOS_gRNA1/2, pIL-1R1_gRNA1/2, pIRGM_gRNA1/2, pCASP1_gRNA1/2, pNLRP1_gRNA1/2, pNLRP3_gRNA1/2, pMyD88_gRNA1/2, and pATG16L1_gRNA1/2 vector, respectively, and was ligated into the KpnI and MluI site of the pEF6-hCas9-Puro vector.

10.1128/mBio.01738-18.5 TABLE S1 Primers used in this study. Information regarding primer names, restriction enzymes, and sequences and the resulting plasmids is shown. Download Table S1, PDF file, 0.1 MB. Copyright © 2018 Bando et al. 2018 Bando et al. This content is distributed under the terms of the Creative Commons Attribution 4.0 International license.

### Generation of gene-targeted human cell lines by CRISPR/Cas9 genome editing.

Human cells were electroporated with the pEF6-hCas9-Puro vector containing target gRNA1/2 using NEPA21 (Nepa Gene). At 24 h postelectroporation, 0.5 to 5 µg/ml puromycin was added for 5 to 10 days to select for cells with stably integrated genes. Cells were plated in limiting dilution in 96-well plates to isolate single-cell clones. To confirm complete target gene deficiency, the expression levels of IDO1, iNOS, IL-1R1, IRGM, CASP1, NLRP1, NLRP3, MyD88, and ATG16L1 protein were analyzed by Western blotting.

### Plasmid construction for generation of knockout T. gondii strains.

Plasmid pSAG1::Cas9-U6::sgUPRT, encoding the Cas9 nuclease (green fluorescent protein [GFP] fusion) under the control of the T. gondii SAG1 promoter, was obtained from addgene (plasmid 54467). The primer sequences are listed in Table S1. The GRA15-targeting CRISPR/Cas9 plasmid (pSAG1::Cas9-U6::sgGRA15-1 or pSAG1::Cas9-U6::sgGRA15-2) was constructed in two steps. First, the overlap-PCR method was used to generate gRNA-expressing plasmids. The U6 promoter from ME49 driving expression of the GRA15-specific single guide RNA (sgRNA) (pgGRA15-1 or pgGRA15-2) was amplified from pSAG1::Cas9-U6::sgUPRT. For first-step PCR, primer pair TgU6_F and GRA15gRNA1-R, primer pair GRA15gRNA1-F and TgU6_R, primer pair TgU6_F and GRA15gRNA2-R, and primer pair GRA15gRNA2-F and TgU6_R were used. For second-step PCR, primer pair TgU6_F and TgU6_R was used and was cloned into the NotI and SacI sites of pBluescript II SK(+) (plasmid 54467). Second, the Cas9-Ds-Red monomer cassette under the control of the SAG1 promoter was cut out of pSAG1::Cas9-U6::sgUPRT (Ds-Red monomer fusion) and ligated into the KpnI and NotI site of the pgGRA15-1 or pgGRA15-2. To generate a construct for deleting the entire coding sequence of GRA15, flanking regions of sequences 5′ outside the sgGRA15-1 regions and 3′ outside the sgGRA15-2 regions were used to surround the GRA15 cassette. To generate a plasmid for insertion of HXGPRT into the GRA15 gene, upstream regions of sgGRA15-1 (663 bp) and downstream regions of sgGRA15-2 (655 bp) were amplified from ME49 genomic DNA by using primers GRA15 targeting 5′_F and GRA15 targeting 5′_R or GRA15 targeting 3′_F and GRA15 targeting 3′_R. These two fragments were ligated into the KpnI and XhoI or BamHI and NotI sites of pHXGPRT vector.

### Generation of gene-targeted T. gondii strains by CRISPR/Cas9 genome editing.

Prugniaud (Pru) T. gondii parasites expressing luciferase were filtered, washed, and resuspended in Cytomix (10 mM KPO_4_, 120 mM KCl, 0.15 mM CaCl_2_, 5 mM MgCl_2_, 25 mM HEPES, 2 mM EDTA). Parasites were mixed with 50 µg of sgGRA15-1 and sgGRA15-2 CRISPR plasmid along with 40 µg of the targeting vector linearized by KpnI and SacI and were supplemented with 2 mM ATP and 5 mM glutathione (GSH). Parasites were electroporated by the use of a Gene Pulser II instrument (Bio-Rad Laboratories). Selection by growth for 14 days in 25 µg/ml mycophenolic acid (Sigma)–25 µg/ml xanthine (Wako) was used to obtain a stably resistant clone. And then parasites were plated in limiting dilution in 96-well plates to isolate single clones. To confirm the disruption of the gene encoding GRA15, we analyzed mRNA of GRA15 from WT and GRA15-KO parasites by quantitative reverse transcription-PCR (RT-PCR). In addition, we observed comparable levels of *in vitro* growth and *in vivo* virulence with respect to each of the mutants and to the parental line.

### Complementation of GRA15 in GRA15-KO T. gondii.

To complement the GRA15-KO parasites, the GRA15 coding region and putative promoter region (1,940 bp upstream of the start codon) were amplified from Pru T. gondii genomic DNA using primers TgGRA15_F and TgGRA15_R (Table S1), subcloned into pCR-Blunt II TOPO (Thermo Fisher), and sequenced. The plasmid was cut by BamHI and PacI, and the fragment containing the gra15 promoter and GRA15 coding region was cloned into the BamHI and PacI sites of the pS1K_DsRed vector, which express DsRed-Express (Clontech) under the control of the T. gondii SAG1 promoter (60). GRA15-KO Pru T. gondii parasites were filtered, washed, and resuspended in Cytomix (10 mM KPO_4_, 120 mM KCl, 0.15 mM CaCl_2_, 5 mM MgCl_2_, 25 mM HEPES, 2 mM EDTA). Parasites were mixed with 50 µg of pS1K_DsRed/empty vector or pSIK_DsRed/gra15-promoter GRA15 expression vector supplemented with 2 mM ATP and 5 mM GSH. Parasites were electroporated by the use of a Gene Pulser II instrument (Bio-Rad Laboratories). After 48 h posttransfection, red fluorescent protein (RFP)-positive parasites were sorted using a FACS AriaIII cell sorter (BD). And then parasites were plated in limiting dilution in 96-well plates to isolate a single clone. To confirm the expression of the gene encoding GRA15, we analyzed mRNA of GRA15 from GRA15-KO parasites with empty or GRA15 expression vectors by quantitative RT-PCR.

### Quantitative RT-PCR.

Total RNA was extracted, and cDNA was synthesized using Verso reverse transcription (Thermo Fisher Scientic). Quantitative RT-PCR was performed with a CFX Connect real-time PCR system (Bio-Rad Laboratories) and a Go-*Taq* real-time PCR system (Promega). The values were normalized to the amount of glyceraldehyde 3-phosphate dehydrogenase (GAPDH) for human cells or to the amount of tubulin for T. gondii in each sample. The primer sequences are listed in Table S1.

### Western blotting.

Cells were lysed in lysis buffer (0.5% Nonidet P-40, 150 mM NaCl, 20 mM Tris-HCl, pH 7.5) containing a protease inhibitor cocktail (Roche) and phosphatase inhibitor cocktail (Nacalai Tesque). The cell lysates were separated by SDS-PAGE and transferred to polyvinylidene difluoride membranes (Immobilon-P; Millipore) and subjected to Western blotting using the indicated antibodies as described previously (61).

### Luciferase assay.

Luciferase activities of total cell lysates were measured as described previously (61). Cells were left untreated or treated with 10 ng/ml IFN-γ and/or 20 ng/ml IL-1α and/or 20 ng/ml IL-1β 24 h before or at the same time as the luciferase-expressing T. gondii infection (multiplicity of infection [MOI], 0.5 to 1). To measure the number of T. gondii parasites, all infected cells were collected for the indicated periods and lysed by the use of 100 μl of lysis buffer (Promega) and sonicated. After centrifugation at 20,000 × *g* at 4°C, the luciferase activity of the supernatant was measured using a dual-luciferase reporter assay system (Promega) and a GloMax 20/20 luminometer (Promega). The percentages of the activities in cytokine-stimulated cells over those in unstimulated cells are shown as relative T. gondii numbers in the figures.

### Measurement of the production of NO_2_.

Cells were cultured in 12-well plates with 10 ng/ml IFN-γ and/or 20 ng/ml IL-1α and/or 10 ng/ml IL-1β for the indicated periods. The concentration of NO in the culture supernatant was measured using NO2/NO3 Assay Kit-FX (Dojindo).

### Measurement of IL-1β levels.

Cells were cultured in 12-well plates and infected with T. gondii for the indicated periods. The concentrations of IL-1β in the culture supernatant were measured by enzyme-linked immunosorbent assay (ELISA) according to the manufacturer’s instructions (eBioscience) using antibodies for IL-1β.

### Immunofluorescence assays.

Huh7 cells were cultured on glass coverslips, infected with T. gondii (MOI = 2) for the indicated time, and fixed in phosphate-buffered saline (PBS) containing 3.7% paraformaldehyde for 10 min at room temperature. Cells were permeabilized with PBS containing 0.002% digitonin for 5 min and then blocked with 8% FBS–PBS for 1 h at room temperature. And then, cells were incubated with the indicated primary antibodies for 1 h at 37°C, followed by incubation with Alexa 488-, Alexa 594-, or Alexa 647-conjugated secondary antibodies (Molecular Probes) and DAPI (4′,6-diamidino-2-phenylindole) for 1 h at 37°C in the dark. Finally, coverslips were mounted onto glass slides with PermaFluor (Thermo Scientific) and analyzed using confocal laser microscopy (FV1200 IX-83; Olympus).

### Coculture experiment.

Primary human hepatocytes, primary mouse hepatocytes, or Huh7 cells were cultured in 12-well plates for 24 h and then washed twice with PBS before coculture. Primary human monocytes or BMDMs or THP-1 cells were plated in 12-well plates and left uninfected or infected with T. gondii (MOI = 1 to 3). At 24 h postinfection, uninfected or infected primary human monocytes, BMDMs, or THP-1 cells or culture supernatant was placed directly on top of the 12-well plates containing the primary human hepatocytes or primary mouse hepatocyte or Huh7 cells in the absence or presence of 10 ng/ml IFN-γ. After 24 to 48 h of treatment with IFN-γ, all cells or culture supernatant was collected and used for each experiment.

### Inhibitor treatment.

Primary human hepatocyte or Huh7 cells were treated with 10 ng/ml IFN-γ and/or aminoguanidine hydrochloride (500 μM) for 24 h and then infected as described above.

### IDO activity assay.

The enzymatic IDO activity was evaluated by the calculation of the kynurenine concentration in the cell culture supernatant as previously described (62). Cells were cultured in 12-well plates and left untreated or treated with 10 ng/ml IFN-γ for 24 h. The concentration of kynurenine in culture supernatant was measured using the Ehrlich reagent method (63). A 70-μl volume of culture supernatant was mixed with 35 μl of 30% trichloroacetic acid, and the mixture was centrifuged at 8,000 × *g* for 5 min. Then, 75 μl of the supernatant was added to an equal volume of Ehrlich reagent (0.8% p-dimethylaminobenzaldehyde–acetic acid) in a 96-well plate, and the absorbance was read at 490 nm. The values were compared with a standard curve with defined concentrations of kynurenine (Sigma-Aldrich).

### Quantitative determination of cell viability (LDH assay).

Cell viability was monitored by measuring lactate dehydrogenase (LDH) activity in the culture supernatant by using a CytoTox 96 non-radio cytotoxicity assay kit (Promega). Huh7 cells were cultured for 24 h and then washed twice with PBS before coculture. THP-1 cells were left uninfected or infected with WT or GRA15-KO Pru T. gondii. At 24 h postinfection, both the uninfected or infected THP-1 cells and the culture supernatant were placed directly on top of the Huh7 cells in the absence or presence of 10 ng/ml IFN-γ. Culture supernatant was collected at the indicated time points and measured by the use of an LDH cytotoxicity colorimetric assay kit according to the manufacturer’s instructions. Culture supernatant of uninfected cells was used as a negative control. Culture supernatant of Triton X-100 (0.1%)-treated cells (the Triton X-100 was used to kill all the cells) was used as a positive control.

### Measurement of parasite numbers by quantitative PCR.

Total DNA was extracted from infected cells by using a DNeasy blood and tissue kit (Qiagen) according to the manufacturer’s instructions and diluted to optimum concentrations for the quantitative PCR (qPCR) assay. The qPCR assays were performed with a CFX Connect real-time PCR system (Bio-Rad Laboratories) using a Go-*Taq* real-time PCR system (Promega). The parasite number was calculated by determination of the amounts of genomic DNA of the SAG1 gene using the standard curve (see Fig. S1F in the supplemental material). The standard curve was established by analysis of the numbers of parasites (ranging from 5 × 10^2^ to 5 × 10^6^ parasites) and of the qPCR cycle numbers of the SAG1 gene DNA.

### Statistical analysis.

All statistical analyses were performed using Prism 7 (GraphPad). All experimental points and *n* values represent averages of results from each of three biological replicates (three independent experiments). The statistical significance of differences in mean values was analyzed by using an unpaired two-tailed Student's *t* test. *P* values of less than 0.05 were considered to be statistically significant.
