# Supplementary figures and images for: Inducible Nitric Oxide Synthase Is a Key Host Factor for Toxoplasma GRA15-Dependent Disruption of the Gamma Interferon-Induced Antiparasitic Human Response
Source: mBio. 2018 Oct 9;9(5):e01738-18. doi: 10.1128/mBio.01738-18 (PMC6178625; doi:10.1128/mBio.01738-18)

Figure S1\_Bando et al.

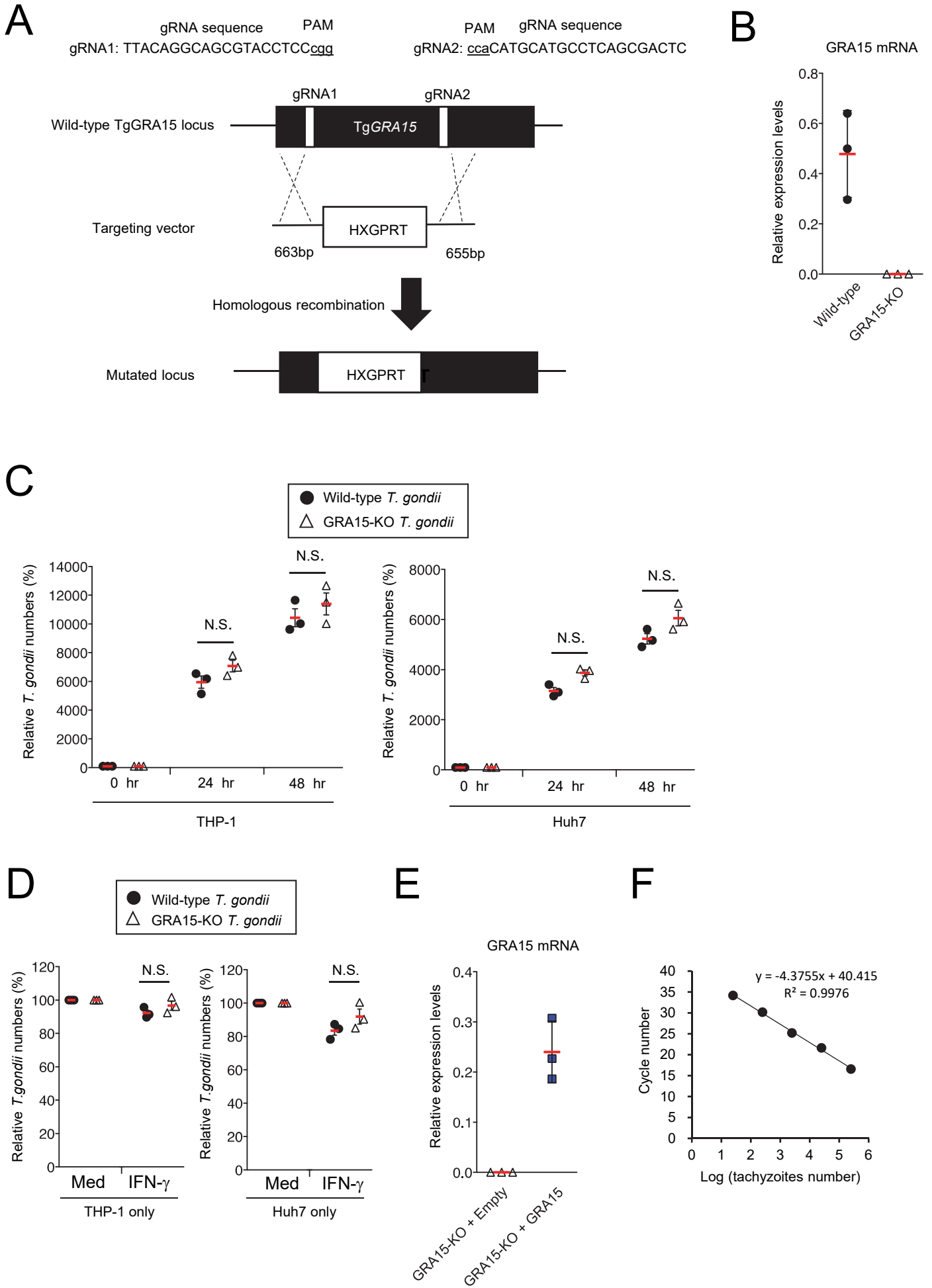

Supplement: FIG S1 [file mbo005184099sf1.pdf]

Figure S2\_Bando et al.

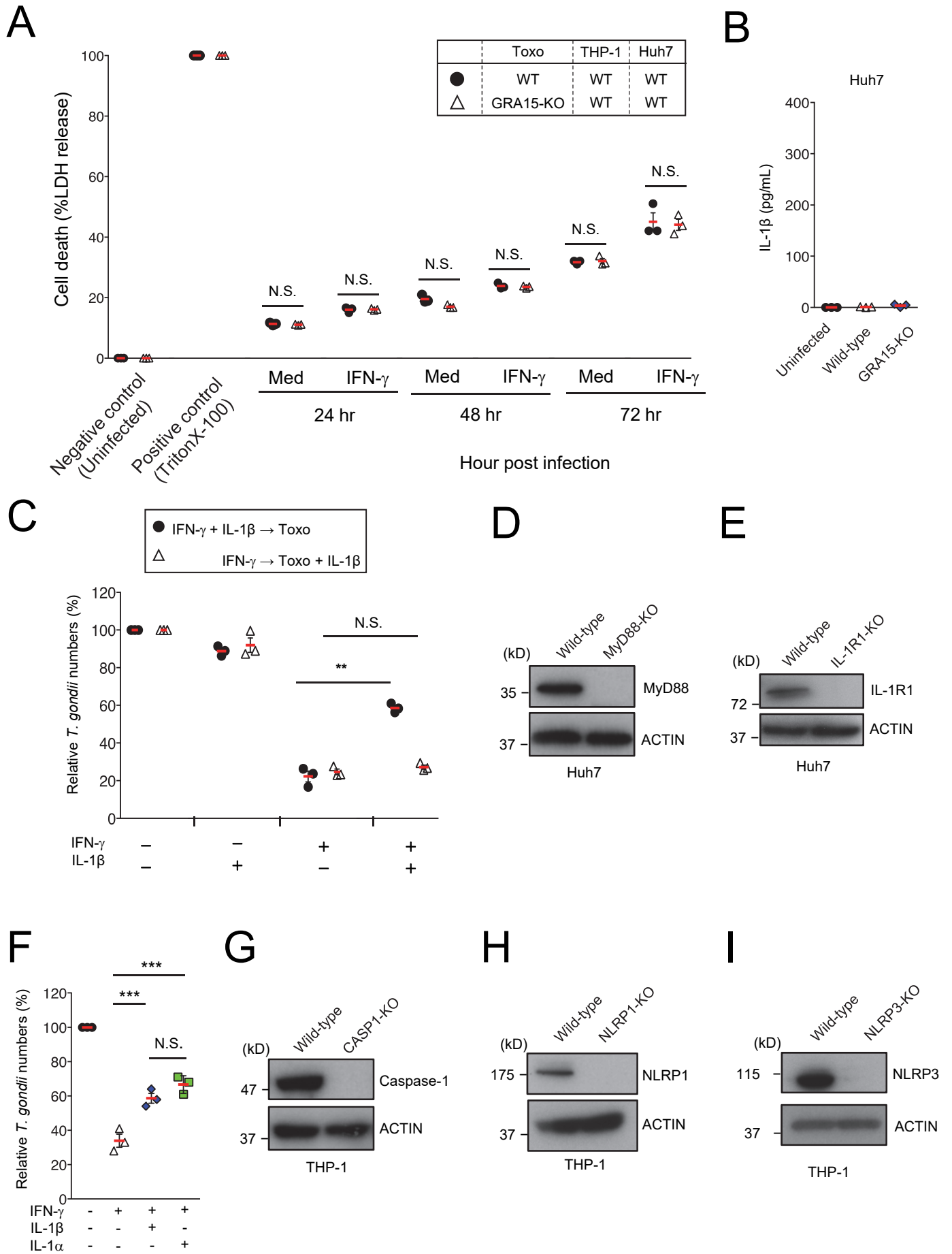

Supplement: FIG S2 [file mbo005184099sf2.pdf]

Figure S3\_Bando et al.

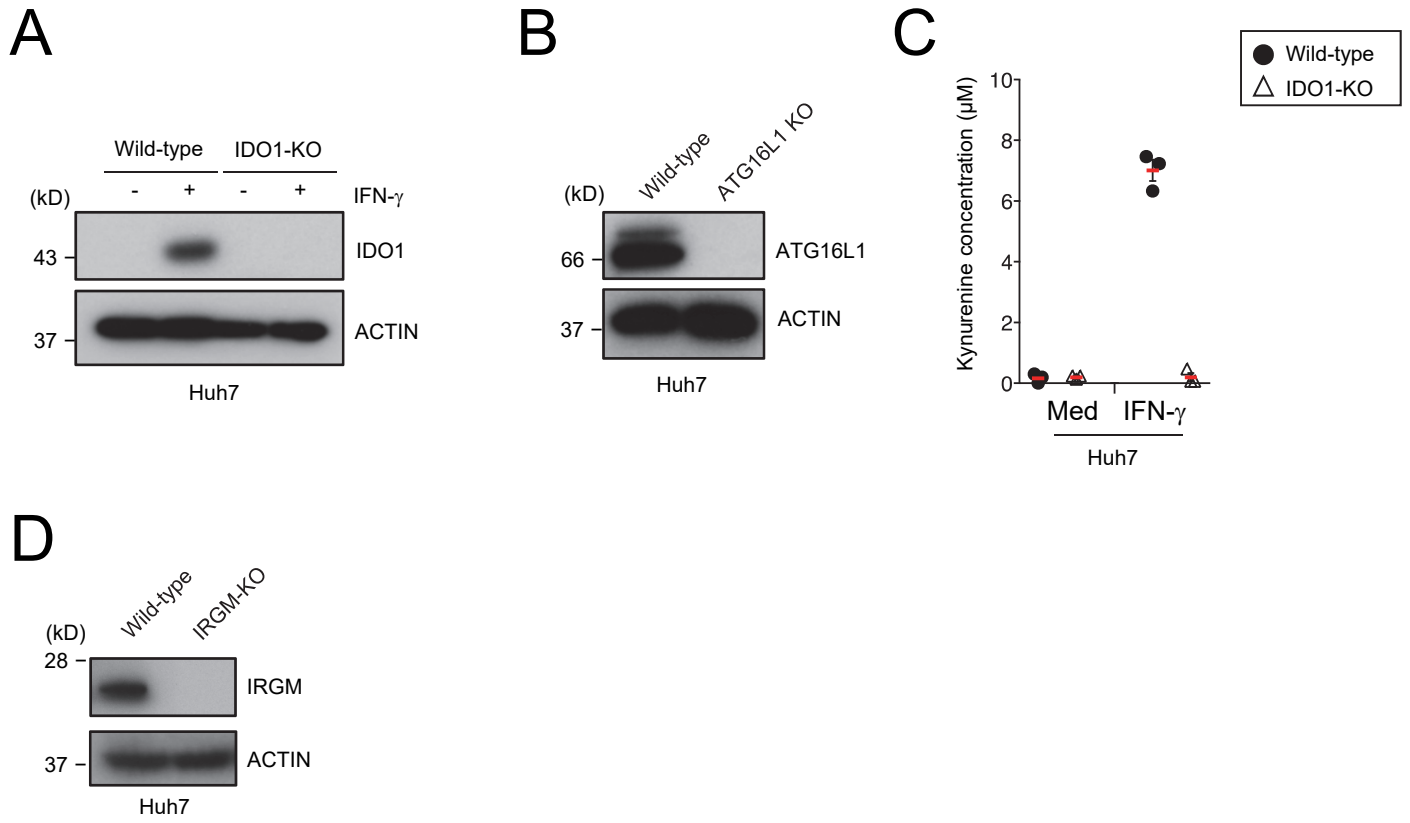

Supplement: FIG S3 [file mbo005184099sf3.pdf]

Figure S4\_Bando et al.

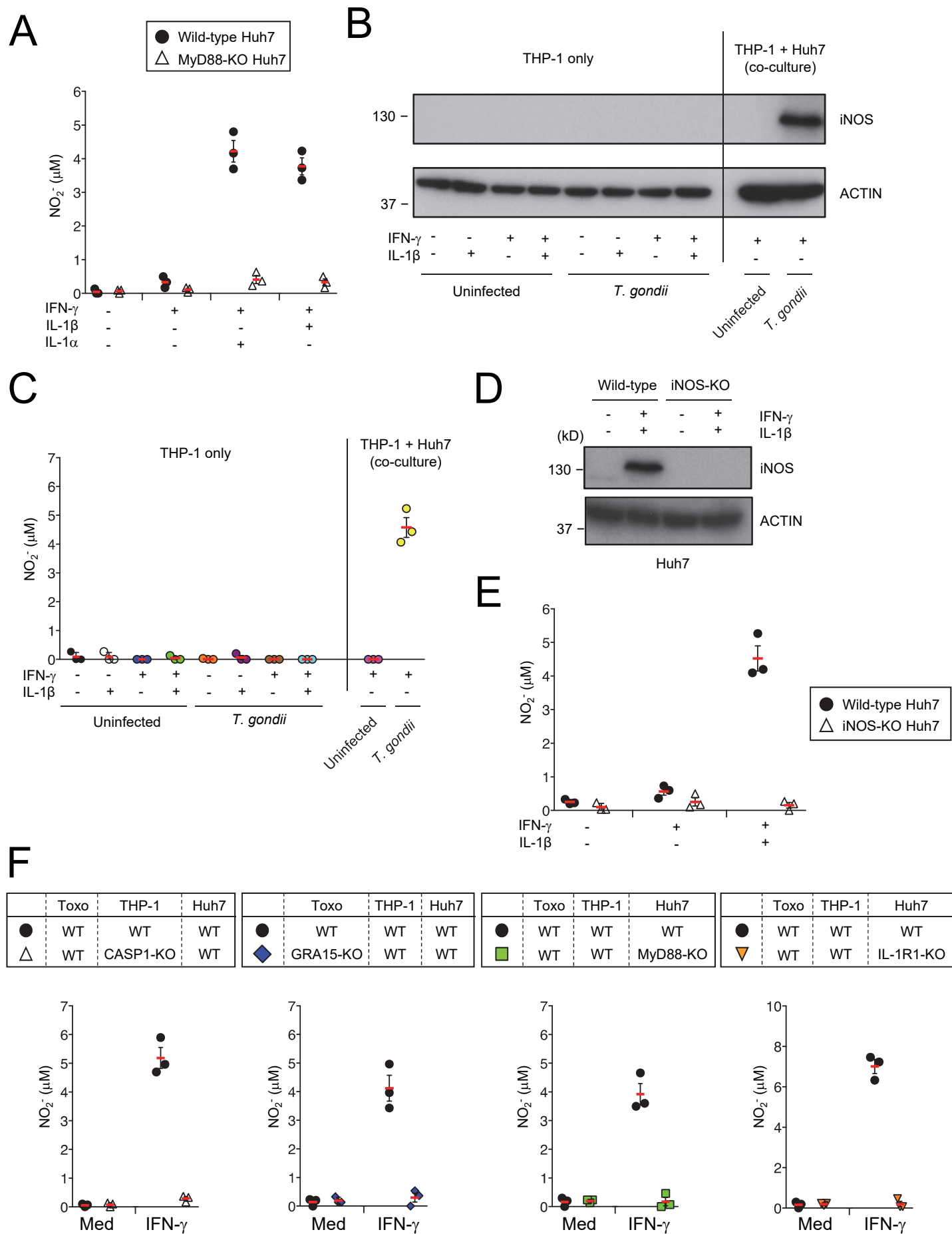

Supplement: FIG S4 [file mbo005184099sf4.pdf]
