# Supplementary material for: Inducible Nitric Oxide Synthase Is a Key Host Factor for Toxoplasma GRA15-Dependent Disruption of the Gamma Interferon-Induced Antiparasitic Human Response
Source: mBio. 2018 Oct 9;9(5):e01738-18. doi: 10.1128/mBio.01738-18 (PMC6178625; doi:10.1128/mBio.01738-18)
Supplement: TABLE S1 [file mbo005184099st1.pdf]

Table S1_Bando et al.

Table S1. Primers used in this study.

Primer name	enzyme	sequence	Resulting plasmids and descriptions
IDO1_gRNA1_F	—	5’-CACCGagttctgggatgcatcacca-3’	IDO1 gRNA1
IDO1_gRNA1_R	—	5’-AAACtggtgatgcatcccagaactC-3’	IDO1 gRNA1
IDO1_gRNA2_F	—	5’-CACCGctgccaactctccaagaaac-3	IDO1 gRNA2
IDO1_gRNA2_R	—	5’-AAACgtttcttggagagttggcagC-3’	IDO1 gRNA2
iNOS_gRNA1_F	—	5’- CACCGccttgtgcttgaggtggcca -3’	iNOS gRNA1
iNOS_gRNA1_R	—	5’- AAACtggccacctcaagcacaaggC -3’	iNOS gRNA1
iNOS_gRNA2_F	—	5’- CACCGcaccaacagccccacattcc -3’	iNOS gRNA2
iNOS _gRNA2_R	—	5’- AAACggaatgtggggctgttggtgC-3’	iNOS gRNA2
IRGM_gRNA1_F	—	5’-CACCGagactctgaagatagtgtcc-3’	IRGM gRNA1
IRGM_gRNA1_R	—	5’-AAACggacactatcttcagagtctC-3’	IRGM gRNA1
IRGM_gRNA2_F	—	5’-CACCGaaacacaggacatgagggta-3’	IRGM gRNA2
IRGM_gRNA2_R	—	5’-AAACtaccctcatgtcctgtgtttC-3’	IRGM gRNA2
IL-1R1_gRNA1_F	—	5’-CACCGagctctactgatttcttctc-3’	IL-1R1 gRNA1
IL-1R1_gRNA1_R	—	5’-AAACgagaagaaatcagtagagctC-3’	IL-1R1 gRNA1
IL-1R1_gRNA2_F	—	5’-CACCGcttaacccaaatgaacacaa-3’	IL-1R1 gRNA2
IL-1R1_gRNA2_R	—	5’-AAACttgtgttcatttgggttaagC-3’	IL-1R1 gRNA2
CASP1_gRNA1_F	—	5’-CACCGtagaagaagctcaaaggata-3’	CASP1 gRNA1
CASP1_gRNA1_R	—	5’-AAACtatcctttgagcttcttctaC-3’	CASP1 gRNA1
CASP1_gRNA2_F	—	5’-CACCGgtgtggtttaaagattcagt-3’	CASP1 gRNA2
CASP1_gRNA2_R	—	5’-AAACactgaatctttaaaccacacC-3’	CASP1 gRNA2
MyD88_gRNA1_F	—	5’-CACCGgaccgcgctggcggaggaga-3’	MyD88 gRNA1
MyD88_gRNA1_R	—	5’-AAACtctcctccgccagcgcggtcC-3’	MyD88 gRNA1
MyD88_gRNA2_F	—	5’-CACCGctgtagacagcagtgtccca-3’	MyD88 gRNA2
MyD88_gRNA2_R	—	5’-AAACtgggacactgctgtctacagC-3’	MyD88 gRNA2
NLRP1_gRNA1_F	—	5’-CACCGcgccggcaattcatggatcc-3’	NLRP1 gRNA1
NLRP1_gRNA1_R	—	5’-AAACggatccatgaattgccggcgC-3’	NLRP1 gRNA1
NLRP1_gRNA2_F	—	5’-CACCGtgggggagaggccagctgta-3’	NLRP1 gRNA2
NLRP1_gRNA2_R	—	5’-AAACtacagctggcctctcccccaC-3’	NLRP1 gRNA2
NLRP3_gRNA1_F	—	5’-CACCGgcacccgctgcaagctggcc-3’	NLRP3 gRNA1
NLRP3_gRNA1_R	—	5’-AAACggccagcttgcagcgggtgcC-3’	NLRP3 gRNA1
NLRP3_gRNA2_F	—	5’-CACCGccccgtgagtcccattaaga-3’	NLRP3 gRNA2
NLRP3_gRNA2_R	—	5’-AAACtcttaatgggactcacggggC-3’	NLRP3 gRNA2
ATG16L1_gRNA1_F	—	5’-CACCGtctcggagcaactgaggcgc-3’	ATG16L1 gRNA1
ATG16L1_gRNA1_R	—	5’-AAACgcgcctcagttgctccgagaC-3’	ATG16L1 gRNA1
ATG16L1_gRNA2_F	—	5’-CACCGggagctggtcaccagatgga-3’	ATG16L1 gRNA2
ATG16L1_gRNA2_R	—	5’-AAACtccatctggtgaccagctccC-3’	ATG16L1 gRNA2
DsRed-Monomer_F	BamHI	5’-GGATCCGACAACACCGAGGACGTCATCAAG-3’	pSAG1::Cas9-U6::sgUPRT (Ds-Red monomer fusion)
DsRed-Monomer_R	PacI	5’-TTAATTAACTAGGACTGGGAGCCGGAGTGGCGGGCCTCGGCGTG-3’	pSAG1::Cas9-U6::sgUPRT (Ds-Red monomer fusion)
TgU6_F	NotI	5’-GCGGCCGCCACCGCGGTGGAGCTCAAGTAAGCAGAAGCACGCTG-3’	pgGRA15-1, pgGRA15-2, pgTgIST-1, pgTgIST-2
TgU6_R	SacI	5’-GAGCTCAAAAAAGCACCGACTCGGT-3’	pgGRA15-1, pgGRA15-2, pgTgIST-1, pgTgIST-2
GRA15gRNA1-F	—	5’-TTACAGGCAGCGTACCTCCCGTTTTAGAGCTAGAAATAGCAAGT-3’	pgGRA15-1
GRA15gRNA1-R	—	5’-GGGAGGTACGCTGCCTGTAAAACTTGACATCCCCATTTACCAGA-3’	pgGRA15-1
GRA15gRNA2-F	—	5’-GAGTCGCTGAGGCATGCATGGTTTTAGAGCTAGAAATAGCAAGT-3’	pgGRA15-2
GRA15gRNA2-R	—	5’-CATGCATGCCTCAGCGACTCAACTTGACATCCCCATTTACCAGA-3’	pgGRA15-2
GRA15 targeting 5’_F	KpnI	5’-GGTACCTGAAACACCACATACCACACAAC-3’	pHXGPRT-GRA15-5’
GRA15 targeting 5’_R	XhoI	5’-CTCGAGGGTACGCTGCCTGTAATTCTCCT-3’	pHXGPRT-GRA15-5’
GRA15 targeting 3’_F	BamHI	5’-GGATCCGCATGCCTCAGCGACTCCCTCGC-3’	pHXGPRT-GRA15-3’
GRA15 targeting 3’_R	NotI	5’-GCGGCCGCGTTTTCTGTTCTGGGTGGTCC-3’	pHXGPRT-GRA15-3’
TgGRA15_F	BamHI	5’-GGATCCGCGGCCGCCGATGGTCTCACTAGGGAACTGCGTCGTCGTGAGTA-3’	pSIK_DsRed/gra15-promoter GRA15
TgGRA15_R	PacI	5’-TTAATTAATCATGGAGTTACCGCTGATTGTGTGTC-3’	pSIK_DsRed/gra15-promoter GRA15
TgSAG1_F	—	5’-CCGGAACAGTACTGATTGTTGTCTTGAG-3’	q-PCR
TgSAG1_R	—	5’-CACAGAGATCCACCTGACTGTGG-3’	q-PCR
hiNOS_F	—	5’-CACAGAGATCCACCTGACTGTGG-3’	Quantitative RT-PCR
hiNOS_R	—	5’-AAGCCGCTGGCATTCCGCACAAA-3’	Quantitative RT-PCR
hGAPDH_F	—	5’-GTCATGGGTGTGAACCATGAGAAG-3’	Quantitative RT-PCR
hGAPDH_R	—	5’-AGTCTTCTGGGTGGCAGTGATG-3’	Quantitative RT-PCR
TgGRA15_F	—	5’-TCACCAGGTACCAACACGTT-3’	Quantitative RT-PCR
TgGRA15_R	—	5’-GGTGCCGTTGGTAGGGTTAA-3’	Quantitative RT-PCR
Tgtublin_F	—	5’-TGTCTGTCGCTGAGATCACCAACT-3’	Quantitative RT-PCR
Tgtublin_R	—	5’-TCTCTTGGTCTTGATGGTCGCAAC-3’	Quantitative RT-PCR
